# High-risk ARGs in environmental *E. coli* from the Taihu Lake watershed

**DOI:** 10.1016/j.isci.2026.117986

**Published:** 2026-10-23

**Authors:** Afeng Wang, Xinyuan Xu, Weiwei Jiang, Bangyi Li, Wenjie Zhu, Qiuping Xiao, Liu Xu, Junhong Sun, Keyu Chen, Yanfeng Yi, Yingying Guo, Xinhua Qiang, Jie He, Hongchang Zhou, Yibin Lin, Xiang Wang

**Affiliations:** 1 School of Medicine, Huzhou Normal University, Huzhou 313000, China; 2 School of Life and Health Sciences, Huzhou College, Huzhou 313000, China; 3 School of Life Sciences, Huzhou Normal University, Huzhou 313000, China; 4 Clinical Laboratory, First People’s Hospital Affiliated to Huzhou Normal University, Huzhou Normal University, Huzhou 313000, China; 5 Infection Disease Department, First People’s Hospital Affiliated to Huzhou Normal University, Huzhou Normal University, Huzhou 313000, China; 6 Key Laboratory for Precise Prevention and Control of Major Chronic Diseases, Huzhou Normal University, Huzhou 31300, China; 7 Zhejiang Key Laboratory of Zero Magnetic Medicine, Affiliated Hangzhou First People’s Hospital, School of Medicine, Westlake University, Hangzhou, Zhejiang, China

**Keywords:** antibiotic resistance genes, *E. coli*, environmental water, Huzhou, Taihu Lake watershed, public health risk

## Abstract

The global antimicrobial-resistance crisis is exacerbated by environmental bacterial reservoirs. This work investigated antibiotic-resistance genes (ARGs) carried by *E. coli* from 118 surface-water samples across the Taihu Lake watershed. We isolated *E. coli* using mTEC agar and *uidA* PCR and screened isolates for 92 ARGs covering 15 resistance classes. Samples from Huzhou showed higher ARG detection rates than samples from other regions, with aminoglycoside, sulfonamide, and glycopeptide resistance genes predominant. Urban sampling sites represented ARG hotspots. Co-occurrence analysis revealed associations between quinolone and aminoglycoside resistance genes, suggestive of horizontal gene transfer. Risk assessment categorized 25.3% of samples as high risk. These observations illustrate human-driven ARG pollution in local aquatic environments and support targeted surveillance and One Health-oriented mitigation strategies.

## Highlights

• High ARG prevalence in Huzhou vs. non-Huzhou waters, driven by anthropogenic activities
• Urban sites (universities, parks) show peak ARG levels, linked to humans and wastewater
• Networks reveal synergy of quinolone (*gyrA*/*parC*) and aminoglycoside (*ant(2″)-I*) via HGT
• Surface waters are ARG hotspots, threatening public health via water/food/contact

## Graphical abstract

**Figure:**
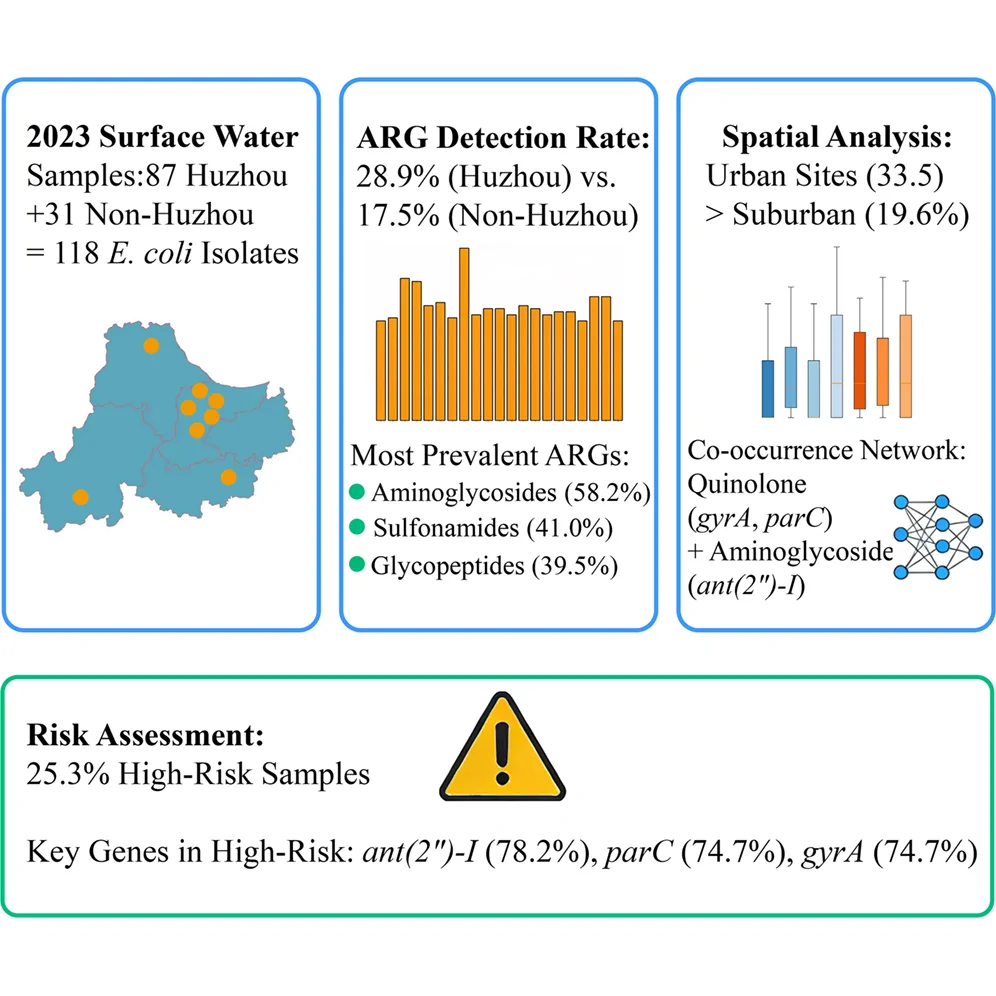

## Teaser

Genetics; Microbial genetics

## Introduction

The global antimicrobial resistance (AMR) crisis poses one of the most significant contemporary threats to public health.^1^ It renders once-treatable infections increasingly difficult or impossible to cure, escalating mortality rates, complicating routine medical procedures, and imposing substantial economic burdens worldwide.^2^^,^^3^ Crucially, this crisis is profoundly amplified by the critical role environmental reservoirs play in the dissemination of resistant bacteria and resistance genes.^4^^,^^5^ Resistant pathogens and genetic determinants shed from human medicine, agriculture (especially intensive livestock production), and industrial waste accumulate in soil, water bodies (rivers, lakes, and oceans), and wastewater systems.^6^^,^^7^ These environmental compartments become vast reservoirs of resistance, functioning not merely as passive depositories but as active hotspots where resistant organisms persist, multiply, and exchange resistance genes through horizontal gene transfer (HGT) mechanisms (e.g., plasmids, and integrons).^8^^,^^9^^,^^10^ This continuous exchange facilitates the spread and evolution of resistance, far beyond their original sources, enabling resistant bacteria to reach new hosts via contaminated water, food, or direct environmental contact.^4^^,^^11^^,^^12^ Effectively combating AMR therefore demands an integrated One Health approach that simultaneously addresses these pervasive environmental pathways alongside human and animal medicine.^13^^,^^14^

Surface waters act as critical reservoirs and dynamic conduits for the environmental dissemination of antibiotic resistance genes (ARGs) and antibiotic-resistant bacteria (ARBs).^4^ These aquatic ecosystems are subject to contamination via multiple pathways, including the discharge of treated effluent from wastewater treatment plants (WWTPs), agricultural runoff laden with antibiotics and manure, and urban runoff transporting pollutants from developed areas.^12^^,^^15^^,^^16^^,^^17^^,^^18^ Consequently, rivers, lakes, and coastal waters accumulate ARGs and ARBs, facilitating their spread across diverse ecosystems and posing a potential risk for human exposure.^19^^,^^20^^,^^21^ To assess the scale and impact of this resistance contamination, indicator organisms, such as fecal coliforms or *E. coli*, are commonly monitored.^22^ While their antibiotic resistance profiles may not directly correlate with those of human pathogens, their presence and resistance patterns serve as valuable proxies.^23^ They primarily indicate fecal contamination levels and, indirectly, signify the potential burden of ARGs entering and circulating within the aquatic environment.^24^

*E*. *coli* holds a unique dual importance, solidifying its position as a critical focus in microbiology and public health.^25^ *E. coli* serves as a well-established fecal indicator bacterium (FIB), making it essential for monitoring microbial safety in water and food supplies.^26^ Its ubiquitous presence in the intestines of warm-blooded animals and throughout the environment further enhances its utility for widespread study and detection.^27^ Beyond its role as an indicator organism, certain pathogenic strains of *E. coli* can cause a wide range of illnesses, from diarrheal diseases to severe conditions such as urinary tract infections, sepsis, and meningitis.^28^ These pathogenic variants carry specific virulence factors, distinguishing them from commensal strains, which are harmless members of the gut microbiota in humans and animals. Its zoonotic potential, with transmission routes involving animals, food, water, and humans, underscores its relevance to both veterinary and human medicine.^29^ Crucially, *E. coli* exhibits well-characterized antibiotic resistance mechanisms and acts as a major reservoir for AMR genes.^30^ Its extensive history as a model organism provides invaluable insights into the evolution and global dissemination of AMR, a critical public health threat. The combination of *E. coli*’s sentinel function, ubiquitous nature, substantial clinical impact, zoonotic links, and pivotal role in AMR research establishes it as an indispensable target for surveillance, research, and clinical management.^31^ Recent studies in the Taihu Lake watershed and other urbanized basins have further confirmed that surface waters act as dynamic conduits for AMR spread, with *E. coli* serving as a key sentinel for tracking resistance.^32^^,^^33^

Studying densely populated urban watersheds is critical due to the intensified pressures exerted by concentrated human activity, industry, and infrastructure on the essential water resources supporting these communities. Huzhou, situated within the intricate network of the Yangtze River Delta and adjacent to the highly stressed Taihu Lake, serves as a vital case study exemplifying these complex dynamics. Nestled amid river networks, low-lying plains, and canals, Huzhou faces significant water quality and management challenges stemming from multiple, interacting pollution sources. Key concerns include non-point source pollution from extensive urbanization generating stormwater runoff and impervious surfaces, diverse industrial discharges concentrated in urban zones, intensive agricultural practices (livestock, aquaculture, and crop cultivation) prevalent in surrounding rural areas contributing nutrient and chemical loads, and the dual pressures of undercapacity or aging wastewater collection and treatment systems failing to capture all domestic sewage and industrial effluents. Understanding the interplay of these factors, including its specific geography, vulnerable water bodies, and the known or potential contributions from urbanization, industry, agriculture, and wastewater infrastructure, is paramount for developing effective strategies to manage Huzhou’s watershed health and mitigate future water resource degradation.

A critical knowledge gap exists regarding the prevalence and distribution of ARGs specifically associated with *E. coli* in the surface waters of Huzhou. Current research lacks comprehensive, localized data detailing which ARGs are carried by this key indicator bacterium within this region’s aquatic environments. This gap hinders our ability to accurately assess the local public health and ecological risks posed by AMR pollution. Generating robust baseline data on *E. coli*-associated ARGs is therefore essential, as it provides the fundamental information required to understand the specific resistance profile within Huzhou’s watersheds, to evaluate the potential risks to human health and ecosystems, and to trace the pathways through which resistant bacteria and genes may transmit and spread in this locality, thereby forming the basis for effective monitoring and management strategies.

This study aims to comprehensively investigate the prevalence and characteristics of antibiotic resistance in *E. coli* within surface waters of Huzhou. The primary objectives are (1) to isolate and identify *E. coli* from diverse sampling locations across the region; (2) to quantify the abundance and characterize the diversity of ARGs harbored specifically by these *E. coli* isolates; (3) to evaluate the spatial distribution of these ARGs, analyzing their relationship with environmental factors and proximity to potential contamination sources; (4) to identify specific associations between the detected ARGs and mobile genetic elements (MGEs), such as integrons and plasmids; and (5) to assess the potential health risks posed by the identified ARG profiles using a quantitative framework, including (a) a risk index (number of detected ARGs per sample) with high-risk thresholds (≥75th percentile), (b) detection frequency analysis of critical ARGs (e.g., last-resort ARGs), and (c) cluster-based source apportionment to identify spatial hotspots of multidrug resistance (MDR).

Collectively, this research seeks to elucidate the environmental reservoir of resistant *E. coli* in Huzhou and understand the drivers and implications of their associated resistance.

## Results

### Overview of sampling and *E. coli* isolation

In 2023, a comprehensive environmental survey investigated surface water ecosystems across China, primarily focusing on natural freshwater bodies. Details of the samples are provided in Tables S1 and S2.

To comprehensively investigate the target region, sampling was conducted across multiple spatial scales. Within Zhejiang Province, the core study area yielded 100 samples from 28 sites, with extensive coverage of Huzhou (including 87 samples from Wuxing District, plus one each from Changxing and Deqing counties) and supplementary collections from other cities (Ningbo: 3; Hangzhou: 2; Jinhua: 1; Quzhou: 1; Dongyang: 3; Jiaxing: 1) (Figures 1A and 1B). To broaden the geographical context, additional 18 samples were gathered: 12 freshwater samples from locations outside Zhejiang (Jiangsu: 1; Shanghai: 3; Beijing: 2; Sichuan: 1; Shaanxi: 1; Guangdong: 1; Fujian: 3) and 6 coastal marine samples from sites including Rizhao, Weihai, Chaozhou, Dongtou, Huludao, and Qingdao (Figure 1A). Specifically targeting the Huzhou watershed as the primary research focus, 87 surface water samples were systematically collected across 19 sites representing all 4 seasons (Figures 1B and 1C). For comparative purposes, 31 non-Huzhou samples (comprising 13 from other Zhejiang cities, 12 from freshwater sites outside Zhejiang, and 6 from coastal marine locations) were incorporated alongside the Huzhou samples, resulting in a comprehensive dataset of 118 samples for analysis (Figures 1A and 1B).

**Figure 1:**
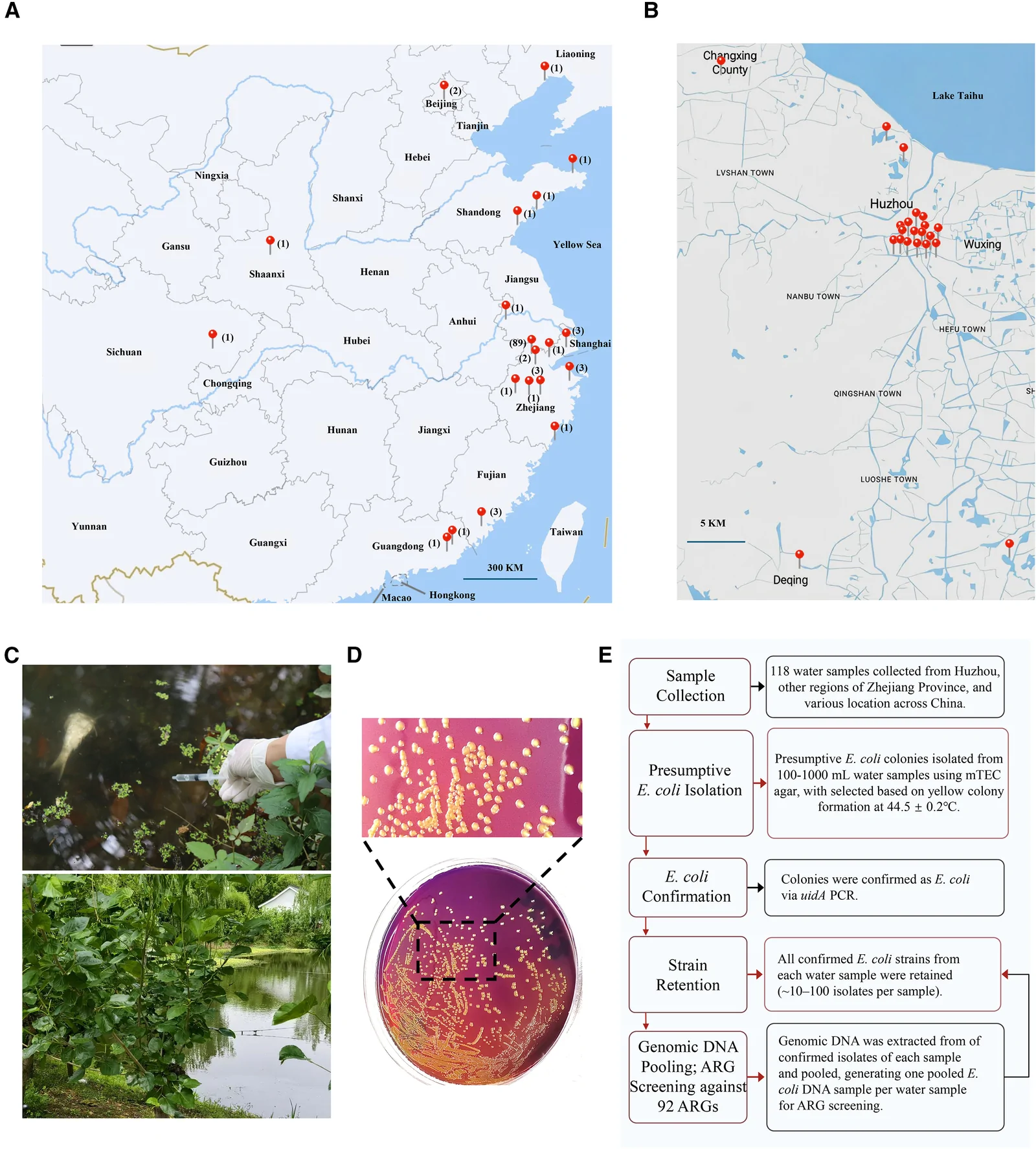
Surface water sampling locations and *E. coli* detection in Huzhou and national context (A) Surface water sampling sites outside the Huzhou region (numbers in parentheses indicate the count of sampling sites per administrative region). (B) Surface water sampling sites within the Huzhou region. (C) Field photographs of representative surface water sampling sites. (D) *E*. *coli* colonies isolated from sampled water using the mTEC culture method. (E) Sampling and isolate identification workflow, including the differentiation of Huzhou and non-Huzhou samples, isolate isolation, *uidA* PCR confirmation, and subsequent ARG analysis. Map scale bars: 5 km (for Huzhou; B) and 300 km (for regional map; A).

Water samples (*n* = 118) were collected from Huzhou, other regions of Zhejiang Province, and various locations across China. Presumptive *E. coli* colonies were isolated from volumes of 100–1000 mL (adjusted according to contamination level) using mTEC agar, with selection based on yellow colony formation at 44.5°C ± 0.2°C (Figure 1D). Colonies were subsequently confirmed as *E. coli* by *uidA* PCR. For each water sample, all confirmed *E. coli* strains (approximately 10–100 colonies per sample) were retained. To evaluate ARG carriage in *E. coli* from each sample, genomic DNA from all confirmed isolates of a given sample was pooled, so that each water sample was represented by a single pooled *E. coli* DNA sample for ARG screening. A schematic of the sampling and processing workflow is presented in Figure 1E.

### *E. coli* abundance and spatiotemporal patterns

*E. coli* was detected across all sampled sites, with an overall recovery success rate of 70% (Figures 2A and 2B). Concentrations exhibited significant spatiotemporal heterogeneity, ranging from <1 CFU (colony-forming unit)/100 mL (detection limit) to 9.3 × 10^4^ CFU/100 mL (Figures 2C and 2D). Geographically, urban-impacted sites (sites HZ9–HZ23 and HZ25–HZ37) consistently demonstrated the highest abundances, with a median concentration of 4.0 × 10^4^ CFU/100 mL, while suburban sites (sites HZ1–HZ4, HZ24, HZ43–HZ66, and HZ73–HZ83) showed significantly lower levels (median: 348.5 CFU/100 mL) (Figure 2D; Table S1). Seasonal variation was pronounced, with peak abundances in summer (median: 4.5 × 10^4^ CFU/100 mL) coinciding with elevated rainfall and temperatures, and the lowest recoveries in winter (median: 265 CFU/100 mL). This widespread prevalence and fluctuating abundance highlight *E. coli* as a persistent component of Huzhou’s surface water microbiome, clearly influenced by anthropogenic activities and seasonal climatic drivers.

**Figure 2:**
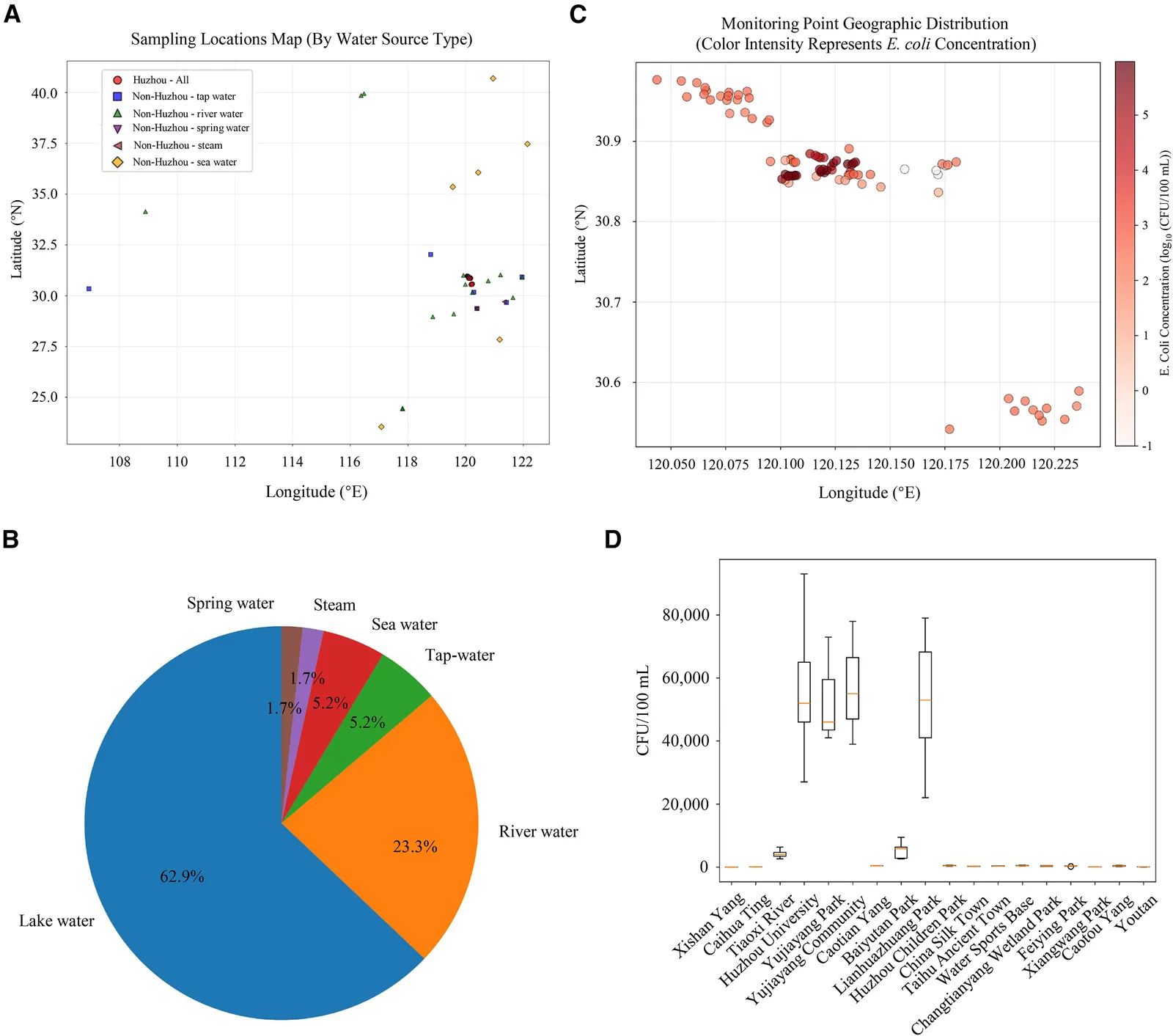
Geographical distribution characteristics of sampling sites and *E. coli* abundance and distribution patterns (A) Geographical distribution of sampling sites by water source type. (B) Proportional distribution of different water sources. (C) Spatial distribution of sampling points with *E. coli* concentration (color intensity indicates log_10_(CFU/100 mL)). (D) Boxplot of *E. coli* abundance (CFU/100 mL) across sampling sites in the Huzhou area. Data are represented as mean ± SEM.

### Huzhou ARGs exhibit higher prevalence and distinct clusters vs. non-Huzhou regions

We first analyzed ARGs harbored by *E. coli* in water samples from Huzhou and non-Huzhou regions (Figures S1 and S2). Significant differences in ARG carriage profiles were observed between the two regions (Figures S3 and S4). PCR screening of *E. coli* isolates revealed a high overall ARG detection rate in Huzhou (mean 28.86% ± 9.91%; Table S3; Figure S1A), significantly exceeding that in non-Huzhou regions (17.50% ± 10.85%; Table S4; Figure S1B). Key findings revealed several notable trends. In the Huzhou region, dominant ARG classes included gentamicin (accounting for 58.24% ± 28.68% of detections, exemplified by *ant(2″)-I*), sulfamethoxazole (41.0% ± 28.46%, such as *sul1*/*2*), and glycopeptides (39.46% ± 27.92%, with *vanA* detected at a notably high rate of 71.28%) (Tables 1 and S5; Figures 3A and S2A). In contrast, polymyxins represented a low-abundance class, showing minimal resistance pressure at just 4.4% ± 9.31% (e.g., *mcr* variants) (Tables 1 and S5; Figures 3A and S2B). Notably, certain unexpected patterns emerged: *vanA*, a glycopeptide resistance gene typically associated with Gram-positive bacteria, and *mecC*, linked to methicillin resistance, were both detected in *E. coli* isolates. However, it is important to emphasize that PCR positivity does not confirm functional resistance, a point further elaborated in the discussion. The relatively high ARG detection rates observed in non-Huzhou water samples were primarily driven by genes associated with specific antibiotic classes, notably gentamicin (44.09% ± 31.48%), neomycin (30.11% ± 19.92%), streptomycin (29.03% ± 12.39%), and sulfamethoxazole (26.88% ± 27.78%) (Table S5; Figures 3B and S2B). Consistent with the findings in the Huzhou region, polymyxins represented a low-abundance class, showing minimal resistance pressure at just 4.52% ± 9.94% (Table S5).

**Table 1:** Summary of detection rates for different ARG categories in Huzhou, Zhejiang, China

Rank	Category	Number of genes	Average detection rate (%)	Minimum detection rate (%)	Maximum detection rate (%)
1	gentamicin	3	58.24	0.00	100.00
2	sulfamethoxazole	3	41.00	0.00	66.67
3	glycopeptides	3	39.46	0.00	66.67
4	chloramphenicol	2	37.36	0.00	50.00
5	streptomycin	4	37.07	0.00	75.00
6	neomycin	3	35.63	0.00	66.67
7	quinolones	11	35.01	0.00	72.72
8	β-lactams	19	32.19	0.00	66.67
9	MRSA	4	28.16	0.00	50.00
10	macrolides	5	25.52	0.00	80.00
11	tetracycline	20	22.70	0.00	50.00
12	streptogramins	8	21.12	0.00	75.00
13	florfenicol	1	18.39	0.00	100.00
14	linezolid	1	9.20	0.00	100.00
15	colistin	5	4.37	0.00	40.00

**Figure 3:**
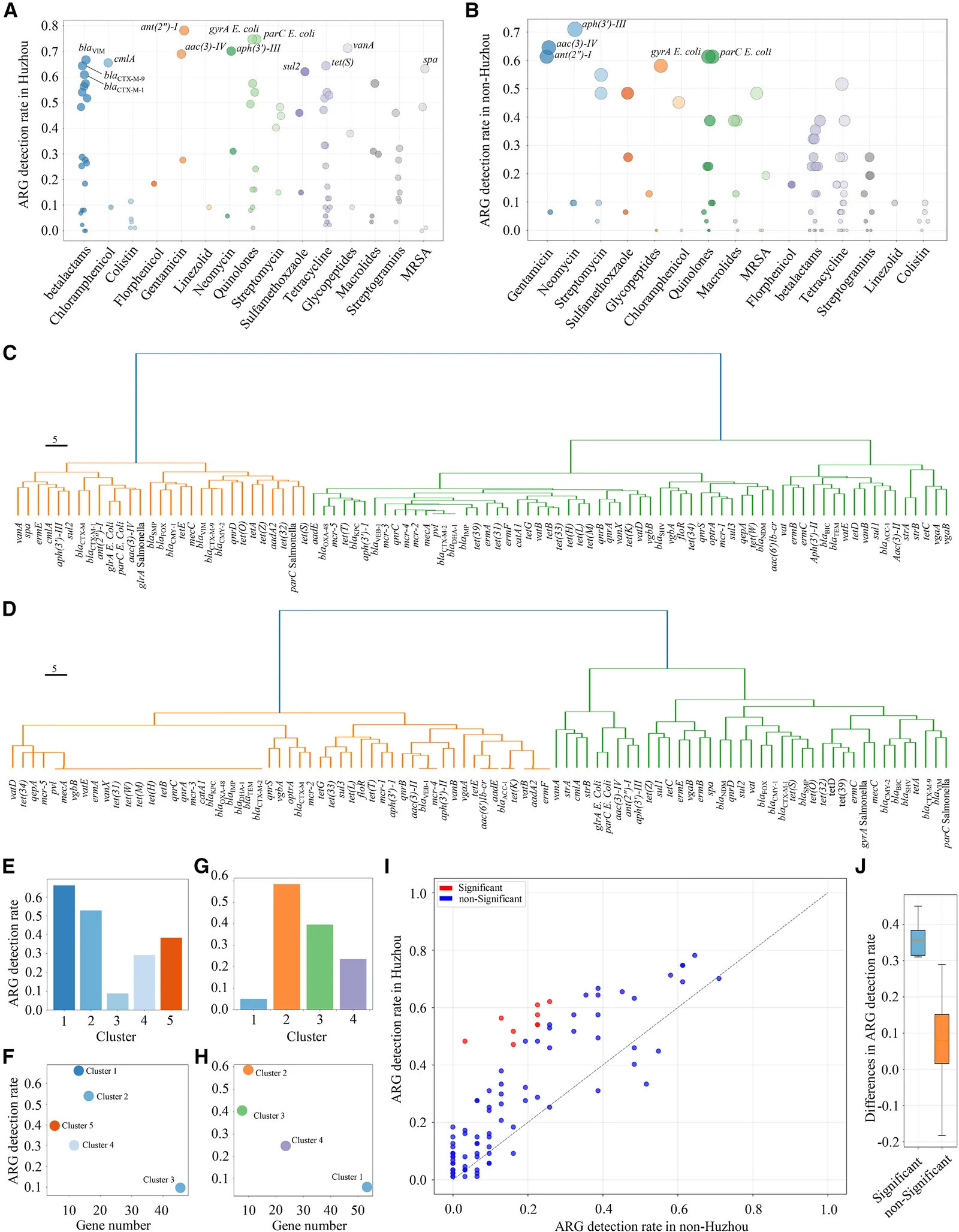
Prevalence and abundance of targeted ARGs (A) Bubble chart of ARG detection rates across antibiotic categories in the Huzhou area. (B) Bubble chart of ARG detection rates across antibiotic categories in the non-Huzhou area. (C) Hierarchical clustering dendrogram of ARGs in the Huzhou region (scale bars indicate distance). (D) Hierarchical clustering dendrogram of ARGs in the non-Huzhou region (scale bars indicate distance). (E) Average ARG detection rates across different clusters in the Huzhou region. (F) Relationship between gene counts and average detection rates of individual ARG clusters in the Huzhou region. (G) Average ARG detection rates across different clusters in the non-Huzhou region. (H) Relationship between gene counts and average detection rates of individual ARG clusters in the non-Huzhou region. (I) Scatterplot comparing ARG detection rates between the two regions; red points denote significant differences after FDR correction, blue points indicate non-significant differences, and the diagonal line represents equal detection rates. (J) Box-and-whisker plot of differences in ARG detection rate for significant and non-significant groups. Boxes show 25th–75th percentiles, central horizontal lines denote medians, and whiskers span values within 1.5 × interquartile range.

We further performed clustering analysis on water samples from different regions and their associated ARGs based on per-sample ARG detection rates (Figures S5 and S6). Hierarchical clustering of ARGs detected in the Huzhou region delineated 5 distinct clusters (Figure 3C). Cluster 1 (13 genes, mean detection rate: 66.2%; e.g., *bla*_CTX-M-1_) and cluster 2 (16 genes, 52.9%; e.g., *bla*_FOX_) were the predominant clusters, exhibiting high average detection rates (Figures 3E and 3F). In contrast, cluster 3 (45 genes, 7.9% mean detection) represented a low-abundance group, likely comprising emerging resistance threats (Figures 3E and 3F). Clusters 4 and 5 (remaining genes) showed intermediate prevalence levels (Figures 3E and 3F). Hierarchical clustering of ARGs from non-Huzhou regions delineated 4 distinct clusters (Figure 3D). Cluster 2 (9 genes, mean detection rate: 58.4%) emerged as a high-risk group, characterized by regionally prevalent genes (Figures 3G and 3H).

Figure 3I presents a scatterplot comparing ARG detection rates between Huzhou and non-Huzhou regions. Red points denote genes with significant differences after false discovery rate (FDR) correction, while blue points indicate non-significant differences. The diagonal dashed line represents equal detection rates between the two regions. Notably, the majority of data points are distributed above the diagonal line, suggesting generally higher ARG detection rates in Huzhou. Figure 3J displays a boxplot comparing the distributions of differences in ARG detection rates between the significant and non-significant groups. The significant group exhibits a consistently higher positive difference (median ∼0.35), whereas the non-significant group shows a wider distribution centered near zero (median ∼0.05), confirming markedly larger detection rate differences in the significant group.

### The spatial distribution of anthropogenic hotspots and gene signatures

The results of the detection rates and distribution characteristics of ARGs in *E*. *coli* from water bodies across different regions of the Huzhou area are presented in Figures S7 and S8. Spatial analysis of 92 ARGs across 7 regions, as detailed in Table 2 and visualized in Figure 4A, uncovered disparities driven by anthropogenic activities. The highest ARG burden was observed in universities (averaging 33.48% ± 33.56% detection) and urban parks (29.08% ± 23.83% average), likely attributable to dense human populations and wastewater inputs in these areas. In contrast, the suburban parks exhibited the lowest burden at 19.56% ± 26.57%, reflecting reduced direct human impact. Intermediate burdens were noted in urban rivers/lakes (26.96% ± 30.73% and 26.96% ± 22.88% with similar rates) and suburban lakes (24.74% ± 21.64%), while communities showed moderate levels. Regarding variability, universities, urban rivers, and urban parks displayed the widest interquartile ranges (IQRs) and longest whiskers (reaching 1.0, ∼0.8, and ∼0.8, respectively), indicating highly variable ARG detection. In contrast, suburban parks demonstrated a more stable ARG prevalence distribution (compact IQR).

**Table 2:** Proportion of samples and average detection rate of ARGs across different geographical regions in Huzhou, Zhejiang, China

Geographical region	Proportion of samples (%)	Average ARG detection rate (%)
University	5.7	33.5
Urban park	31.0	31.0
Urban river	13.8	27.9
Urban lake	21.8	27.9
Community	3.4	25.0
Suburban lake	18.4	22.0
Suburban park	5.7	19.6

**Figure 4:**
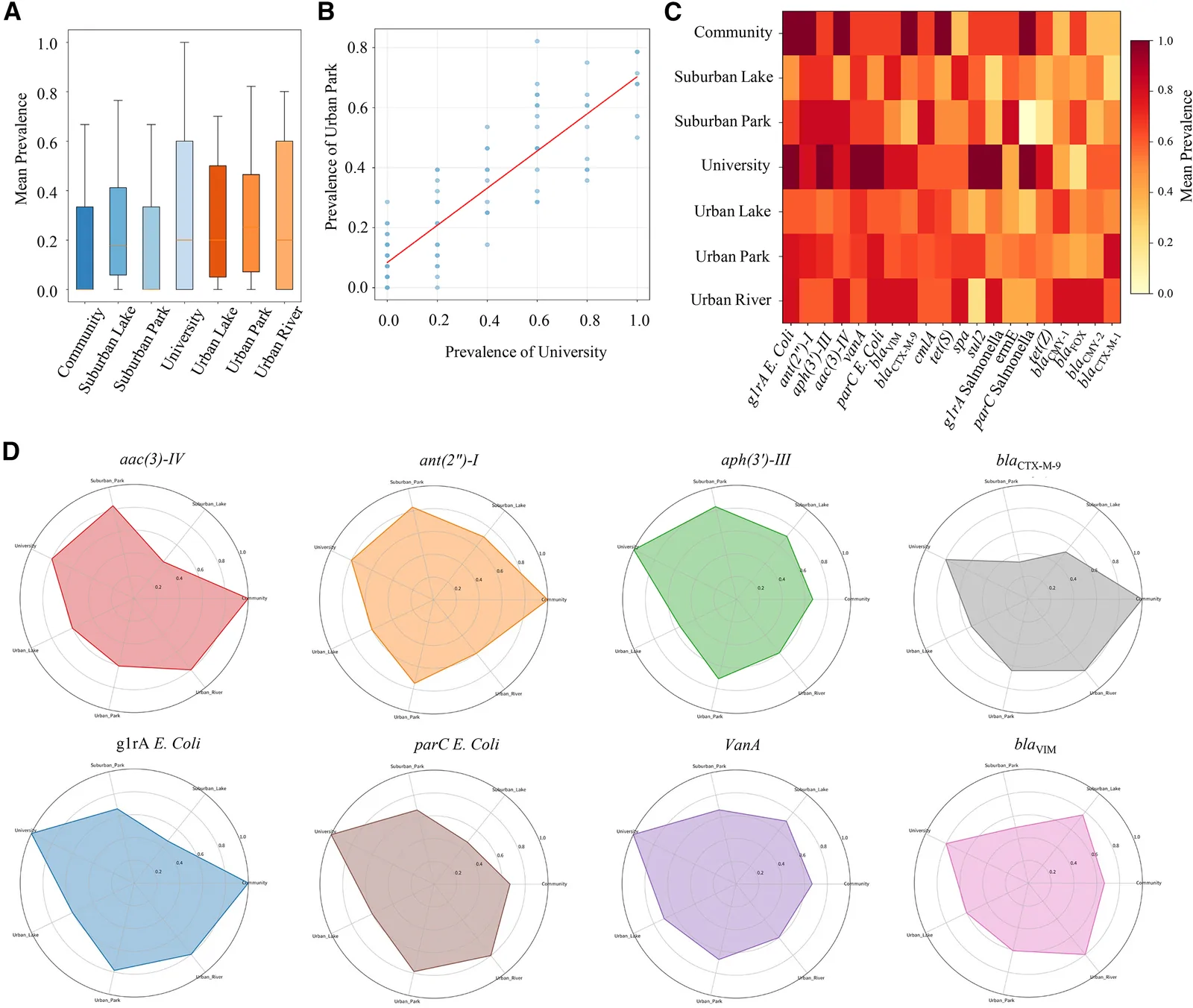
Spatial patterns of ARG distribution (A) Bar chart showing mean prevalence across different sampling-site communities. Bars represent group means; error bars indicate standard deviation (SD). (B) Scatterplot showing the correlation of ARG prevalence between universities and urban parks (red line indicates the trend). (C) Heatmap of prevalence for the top 20 most frequently detected ARGs across regions. (D) Radar charts of prevalence for selected ARGs.

Figure 4B presents a scatterplot showing the correlation of ARG prevalence between university and urban park, with the red line indicating the trend. The data points exhibited a clear positive correlation, as evidenced by the upward-sloping trend line. As the ARG prevalence at university sites increased (ranging from 0.0 to 1.0), the prevalence at urban park sites correspondingly increased (ranging from 0.0 to ∼0.8). However, the data points were widely dispersed around the trend line, indicating considerable variability in the prevalence matching between the two site categories. The positive correlation suggests that university and urban park sites share similar environmental drivers or sources of antibiotic resistance, implying that ARGs prevalent in one setting are likely to be prevalent in the other. However, the wide dispersion of data points indicates that this relationship is not strictly linear. This variability aligns with the distinct prevalence variabilities observed in Figure 4A (where universities showed high variability and urban parks showed more stable prevalence). It further suggests that while these two site types may exchange resistance genes or face similar anthropogenic pressures, site-specific factors significantly modulate the local ARG abundance.

ARG prevalence distribution was right-skewed: most ARGs were rare (<0.2), but a subset reached 0.7 (Figure S9). Figure 4C presents a heatmap illustrating the mean prevalence of the top 20 most frequently detected ARGs across the 7 sampling regions. Communities had high levels (e.g., *gltA* in *E. coli*, *ant*(2″)-I), suburban lakes moderate, universities enriched in *gyrA* and *parC* of *E. coli*, urban rivers in *bla*_CMY_ groups/*bla*_FOX_, and urban parks in *gyrA*/*bla*_CTX-M-1_. In contrast, suburban lakes and urban lakes displayed relatively lower prevalence (lighter orange/yellow shades) across many genes. Notably, specific genes such as *sul2* and *emrE* showed low prevalence (light yellow) in several regions, whereas genes like *gyrA* in *E. Coli*, *parC* in *E. Coli*, and *bla*_CTX-M-9_ demonstrated consistently high prevalence (dark red) across most sites. The heatmap reveals distinct spatial heterogeneity in the prevalence of ARGs. The consistently high prevalence at the university site suggests intense anthropogenic pressures (e.g., high human traffic or specific wastewater discharges) driving the widespread distribution of resistance genes. The varying prevalence patterns of specific genes (e.g., the low prevalence of *sul2* and *emrE* in certain areas) indicate that different ARGs are subject to unique environmental selection pressures and ecological constraints. Furthermore, the broad detection of key genes (e.g., *bla*_CTX-M-9_ and *gyrA* in *E. Coli*) across all regions highlights the widespread nature of certain antibiotic resistances in urban and suburban environments.

Figure 4D presents radar charts illustrating the prevalence distributions of eight selected ARGs across the seven sampling site categories. The prevalence patterns varied distinctly among the targeted genes (Figures 4D and S10). Specifically, *gyrA* in *E. Coli*, *vanA*, and *aac(3)-IV* exhibited high prevalence at university and suburban park sites. In contrast, *ant(2″)-I*, *bla*_CTX-M-9_, and *parC* in *E. Coli* showed elevated levels primarily in community and urban river sites. Notably, suburban lakes consistently displayed low prevalence across most of the selected ARGs. The distinct radar profiles indicate strong spatial heterogeneity and site-specific distribution patterns of ARGs. The high prevalence of specific genes at university and suburban park sites suggests intense localized anthropogenic pressures and unique environmental selection driving the dissemination of certain resistance determinants. Conversely, the enrichment of other genes (e.g., *bla*_CTX-M-9_) in community and urban river sites points to different sources or ecological constraints. Furthermore, the consistently low prevalence at suburban lakes implies weaker selection pressure or less contamination from resistance sources in this environment.

### Co-occurrence networks and MDR gene synergy

To further evaluate the public health risk posed by concurrent resistance determinants, we investigated the co-occurrence patterns and synergistic potential of MDR genes across all sampled environmental sites, including rivers, urban lakes/parks, and university campuses (Table S7). This analysis revealed that the co-occurrence of MDR genes is a widespread and significant concern. Specifically, examination of ARGs in 87 water samples from the Huzhou region showed that the vast majority of samples harbored multiple ARGs. The detection rate of the 92 tested ARG types varied substantially across sites, ranging from 4.35% at site HZ59 (4 out of 92 ARGs detected) to 61.96% at site HZ27 (57 out of 92 ARGs detected) (Figure 5A; Table S7). These findings confirm that most samples carried substantial ARG loads. Distribution analysis of ARG counts indicated that the majority of the 87 samples clustered within an intermediate range characterized by moderate ARG abundance, while a smaller proportion displayed either very low (<10) or very high (>40) counts (Figure 4B).

**Figure 5:**
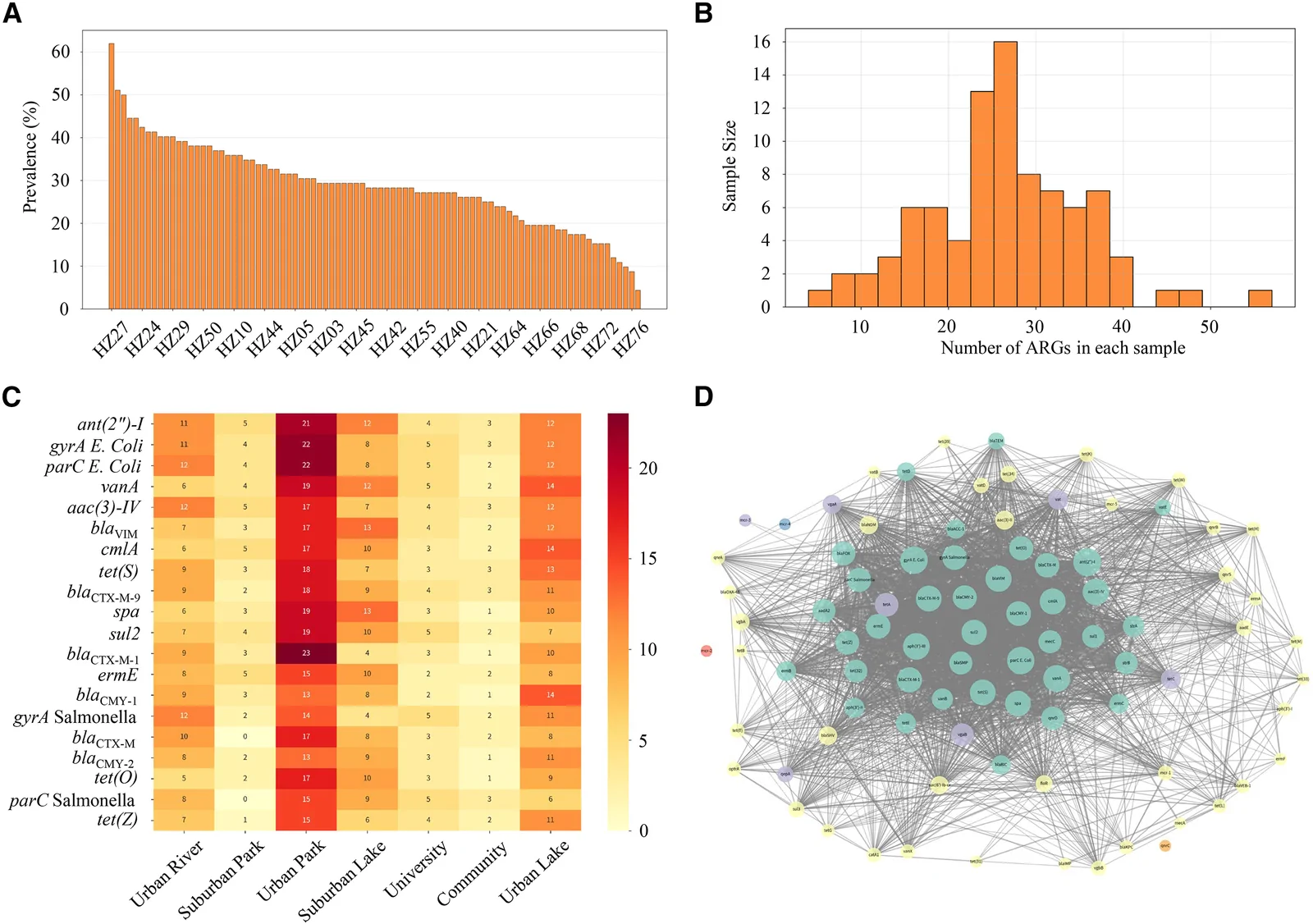
Co-occurrence and diversity of ARGs (A) Prevalence of ARGs in each sample (sorted by prevalence). Bar plot showing the percentage of samples in which individual ARGs were detected, with samples ordered by decreasing prevalence of ARGs. (B) Distribution of the number of unique ARGs per sample. Histogram illustrating the frequency of samples containing different quantities of distinct ARGs. (C) Geographical distribution of the top 20 most prevalent ARGs. Heatmap depicting the detection frequency of the 20 most common ARGs across 7 environmental locations (urban rivers, suburban parks, urban parks, suburban lakes, universities, communities, and urban lakes). (D) Co-occurrence network diagram of ARGs. Network visualization where nodes represent ARGs and edges denote significant co-occurrence relationships, with node size proportional to ARG prevalence and edge thickness indicating the strength of association.

This observation underscores that most water samples collected from Huzhou contained multiple ARGs. Analysis of the detection data of the top 20 ARGs with higher detection rates in different geographical environments revealed that the peak detection of *bla*_CTX-M-1_ occurred in urban parks (23 times), and this environment generally showed a relatively high abundance of ARGs (Figure 5C). In terms of environmental differences, the ARG detection amounts in suburban lakes and universities were mainly at medium to low levels, while the detection amounts in other environments such as suburban parks and communities, though fluctuating, remained overall lower than those in urban parks (Figure 5C). Further gene-specific pattern analysis revealed that multiple ARGs, including *ant(2″)-I*, *E*. *coli gyrA*, and *vanA*, exhibited significantly different distributions across various environments, confirming the spatial heterogeneity of MDR gene contamination (Figure 5C).

Through the analysis of co-occurrence patterns of 92 ARGs in 87 samples from the Huzhou region, this study revealed the distribution patterns and synergistic relationships of ARGs in the environment (Figures 5D and S11). A total of 92 drug resistance genes were analyzed for their distribution across 87 samples, of which 89 genes were detected in at least one sample (with 3 genes not detected), and on average, each gene was found in 25.96 samples. The most prevalent gene was *ant(2″)-I*, which was detected in 68 samples (Table S7). The gene co-occurrence network illustrated the synergistic occurrence patterns among genes, where node size reflected gene prevalence and edge thickness indicated co-occurrence strength (Figure 5D). Network analysis revealed multiple closely associated gene clusters. Among the key co-occurrence relationships, the top five strongest co-occurrence relationships and their mutual detection rates were as follows (Table 3; Figure 5F): *gyrA* in *E. coli* and *parC* in *E. coli* co-occurred 61 times; *ant(2″)-I* and *gyrA* in *E. coli* co-occurred 55 times; *ant(2″)-I* and *parC* in *E. coli* also co-occurred 55 times; *aac(3)-IV* and *gyrA* in *E. coli* co-occurred 52 times; *aph(3′)-III* and *gyrA* in *E. coli* similarly co-occurred 52 times. The study concluded that in the Huzhou environment, high-prevalence ARGs such as *ant(2″)-I*, *gyrA* in *E. coli*, and *parC* in *E. coli* have been widely present and deeply integrated into local microbial communities, becoming important components thereof. Among these, *gyrA* in *E. coli* and *parC* in *E. coli* exhibited near-complete co-occurrence characteristics, and this strong synergistic relationship suggests that they may share horizontal transfer mechanisms or be driven by similar selective pressures.

**Table 3:** Top 5 strongest co-occurrence relationships of ARGs and their mutual detection rates in Huzhou, Zhejiang, China

Rank	Co-occurrence pair	Co-occurrence count	Detection rate (entity 1 → entity 2)	Detection rate (entity 2 → entity 1)
1	*glrA E. Coli* ↔ *parC E. Coli*	61	93.8% (*parC E. Coli* in samples with *glrA E. Coli*)	93.8% (*glrA E. Coli* in samples with *parC E. Coli*)
2	*ant(2″)-I* ↔ *glrA E. Coli*	55	80.9% (*glrA E. Coli* in samples with *ant(2″)-I*)	84.6% (*ant(2″)-I* in samples with *glrA E. Coli*)
3	*ant(2″)-I* ↔ *parC E. Coli*	55	80.9% (*parC E. Coli* in samples with *ant(2″)-I*)	84.6% (*ant(2″)-I* in samples with *parC E. Coli*)
4	*aac(3)-IV* ↔ *glrA E. Coli*	52	86.7% (*glrA E. Coli* in samples with *aac(3)-IV*)	80.0% (*aac(3)-IV* in samples with *glrA E. Coli*)
5	*aph(3′)-III* ↔ *glrA E. Coli*	52	85.2% (*glrA E. Coli* in samples with *aph(3′)-III*)	80.0% (*aph*(*3′)-III* in samples with *glrA E. Coli*)

### Risk assessment of high-risk samples, critical genes, and clusters

To quantify health risks, we applied a three-component framework: (1) a risk index (number of detected ARGs per sample) to stratify samples by burden, (2) detection frequency analysis of ARGs to identify high-risk genes, and (3) cluster analysis to map spatial heterogeneity of risk.

The results are based on detection data of 92 ARGs from 87 samples in the Huzhou area. The risk index of the samples (i.e., the number of detected drug-resistant genes) ranged from 4 to 57, with a mean of 26.6 (Figure 6A). Specifically, the lowest-risk sample carried 4 drug-resistant genes, the median was 26, the highest-risk sample had 57, and the 75th percentile was 32.5. The results indicate significant variations in risk among the samples, with some carrying a number of drug-resistant genes far above the average. A total of 22 samples (25.3% of the total) had a risk index at or above the 75th percentile (32.5) and require special attention (Figure 6B). The analysis of detection frequencies across all 92 drug-resistant genes revealed a distribution spanning from 0 to 78.2 percent, with an average frequency of 28.9 percent (Figure 6C). It is worth noting that the gene with the highest frequency was *ant(2″)-I*, which exhibited a detection frequency as high as 78.2 percent. High frequency genes, defined as those with a detection frequency exceeding 70 percent, included *gyrA* and *parC* of *E. Coli*, both at 74.7 percent, as well as the *vanA* gene at 71.3 percent. Genes falling within the medium frequency range of 50–70 percent included *aph(3′)-III*, *aac(3)-IV*, *bla*_VIM_, and others (Figure 6D).

**Figure 6:**
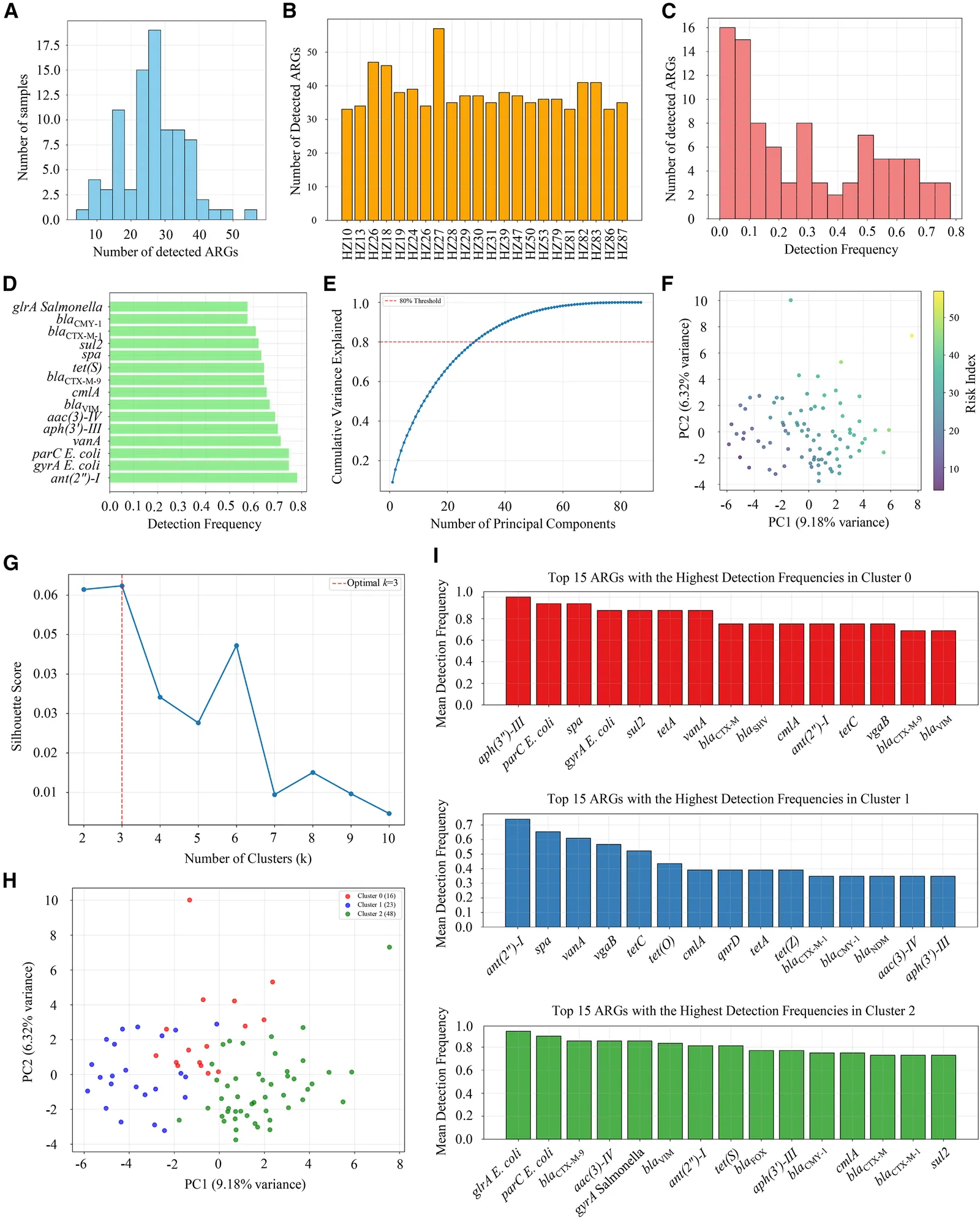
Risk assessment and preliminary source apportionment (A) Distribution of the number of detected ARGs per sample. (B) Number of detected ARGs across individual samples. (C) Distribution of ARG detection frequencies. (D) Top 15 ARGs with the highest detection frequencies. (E) Cumulative variance explained by PCA components. (F) PCA score plot of samples based on the first two principal components (color intensity represents the risk index). (G) Silhouette score analysis for different numbers of clusters (*k*). (H) Sample clustering results with *k* = 3. (I) Top 15 ARGs with the highest detection frequencies in each cluster (clusters 0, 1, and 2).

For preliminary source apportionment, we used principal component analysis (PCA)^34^ as an exploratory tool to visualize broad patterns of ARG distribution across samples. PCA was applied to binary presence/absence data of 92 ARGs (detection vs. non-detection), without transformation—an approach suitable for hypothesis generation but not inferential statistics, given the high correlation and binary nature of the data. The first 5 principal components cumulatively explained 29.2% of the variance (Figure 6E), indicating that sample differences are complex and not fully captured by a few components. The PCA scatterplot (Figure 6F) revealed a tentative clustering trend, with color intensity reflecting the risk index (number of detected ARGs per sample). Importantly, PCA was supplemented with *k*-means cluster analysis (silhouette coefficient-optimized, *k* = 3), a non-parametric method more robust to binary data, to validate spatial heterogeneity (see later). Clustering analysis identified three optimal clusters using silhouette coefficient analysis (0.0623). The sample distribution was 16 in cluster 0, 23 in cluster 1, and 48 in cluster 2 (Figures 6G and 6H). Cluster 0 comprised 16 samples with an average risk index of 32.98, representing the highest risk group and carrying multiple resistance genes. Cluster 1 consisted of 23 samples with an average risk index of 18.05, indicating a low-risk group with fewer resistance genes. Cluster 2 included 48 samples with an average risk index of 32.68, classifying it as a high-risk group that also carried multiple resistance genes. Distinct differences in ARG profiles were observed among clusters, and cluster gene characteristic maps illustrated the most common ARGs in each category (Figure 6I).

## Discussion

This study presents the first comprehensive characterization of ARG prevalence, abundance, spatial distribution, and temporal dynamics within *E*. *coli* populations inhabiting the diverse surface waters of Huzhou, China. Our findings reveal a landscape significantly shaped by anthropogenic pressures and environmental drivers, with important implications for environmental and public health risk assessment.

The analysis of targeted ARGs in *E. coli* isolates from Huzhou and non-Huzhou regions reveals critical insights into the environmental resistome and its anthropogenic drivers. The higher average ARG detection rate in Huzhou (28.9%) vs. non-Huzhou regions (17.5%) suggests localized anthropogenic activities (e.g., wastewater and agriculture) may contribute to ARG accumulation.^4^^,^^35^^,^^36^

The distribution of ARGs in Huzhou reveals distinct selective pressures driven by antibiotic usage patterns and environmental persistence. Aminoglycosides (58.2%), sulfonamides (41.0%), and glycopeptides (39.5%) emerged as the most prevalent classes, reflecting their common application in both human medicine and agriculture, which likely exerts sustained selection pressure.^37^ In contrast, polymyxins (e.g., colistin)—a last-resort antibiotic—were rarely detected (4.4%), suggesting limited regional use and reduced current resistance pressure.^38^ Notably, this pattern aligns with the high detection rates of specific ARGs such as *ant(2″)-I* (aminoglycoside) and *vanA* (vancomycin) in local *E. coli* populations, underscoring the environment’s role as a reservoir for these genes. These genes likely originate from anthropogenic sources (e.g., wastewater and agricultural runoff) and persist through HGT, highlighting the need for integrated One Health surveillance despite requiring further validation of their clinical relevance (e.g., transferability to pathogens).

Complementing this distributional insight, clustering analysis of Huzhou’s 92 ARGs identified 5 distinct genetic clusters, offering a nuanced view of diversity. Cluster 1 (high abundance, 66.2% average detection) and cluster 2 (medium-to-high, 52.9%) dominate the landscape, enriched with clinically significant genes like β-lactamases (e.g., *bla*_CTX-M_ groups) and fluoroquinolone markers—consistent with the widespread integration of these resistance traits into local *E. coli*. Meanwhile, cluster 3 (low abundance, 7.9%) comprises less prevalent but potentially emerging threats, necessitating long-term monitoring. Together, these findings emphasize the complex interplay between antibiotic use, environmental reservoirs, and genetic evolution of resistance, calling for continued surveillance to mitigate public health risks.

The spatial patterns of ARG distribution in Huzhou’s surface waters, as revealed in this study, uncover a complex and heterogeneous landscape shaped predominantly by anthropogenic activities, consistent with recent findings in the Taihu Lake watershed and other urbanized regions.^39^^,^^40^ A key finding is the marked regional disparity in ARG detection rates, with urban-associated sites, particularly Huzhou University (33.5% average detection rate) and urban parks, harboring the highest ARG abundance, while suburban parks (19.6%) showed the lowest. This gradient aligns with 2023–2025 studies showing that land-use intensity (e.g., urban density and wastewater inputs) is a primary driver of ARG enrichment in surface waters.^41^^,^^42^ This gradient directly reflects the intensity of human activity, as densely populated areas with complex waste streams such as university campuses and urban green spaces like parks act as hotspots for ARG accumulation, likely due to inputs from sewage, recreational activities, or concentrated human populations.^43^^,^^44^ In contrast, suburban habitats, with reduced direct human impact, exhibit more moderated gene enrichment, suggesting a link between land-use intensity and ARG dissemination.^45^^,^^46^^,^^47^

The study further highlights the role of specific anthropogenic sources in shaping ARG profiles. For instance, urban rivers were enriched in clinically significant β-lactamase genes (e.g., *bla*_CTX-M-1_ and *bla*_FOX_), indicative of wastewater or agricultural runoff contamination, while university sites showed elevated sulfonamide resistance genes (*sul1*, *sul2*, and *sul3*) and plasmid markers, pointing to fecal pollution from dense human communities.^40^^,^^48^^,^^49^ Such site-specific gene signatures reveal that different human activities (e.g., sewage discharge, agricultural practices, and recreational use) exert distinct selective pressures, driving the accumulation of tailored ARG subsets.^50^^,^^51^^,^^52^ This spatial heterogeneity, even at fine intra-urban scales (e.g., variable prevalence within proximate sites like Xishanyang), challenges the assumption of uniform contamination and emphasizes the need for localized surveillance.

The co-occurrence of ARGs, particularly the near-complete synergy between *gyrA* in *E. coli* and *parC* in *E. coli* (quinolone resistance gene) and their strong association with aminoglycoside resistance genes (e.g., *ant(2″)-I*), implicates HGT mechanisms such as conjugation (mediated by plasmids and integrons) and transduction (via bacteriophages).^53^^,^^54^^,^^55^ These findings highlight the role of MGEs—particularly plasmids (e.g., IncF and IncI groups detected in this study) and integrons (class 1 integrons implicated in ARG carriage)—as key drivers of ARG dissemination in this ecosystem. These clusters suggest that resistance genes may be co-located on MGEs, facilitating their spread within microbial communities.^56^^,^^57^^,^^58^ A 2025 genomic study of the dissemination of mobile ARGs further confirmed that such co-occurrence patterns are driven by MGE-mediated HGT in environmental reservoirs,^59^ while 2025 work in urban rivers highlighted the role of plasmids in consolidating MDR cassettes.^60^ The enrichment of such co-occurrence networks in urban hotspots amplifies the risk of MDR cassette dissemination, as these elements can transfer to clinically relevant pathogens, exacerbating the global AMR crisis.

The severity of these findings lies in the identification of critical resistance genes in environmental reservoirs. Urban sites harbored genes conferring resistance to last-resort antibiotics, including carbapenemases (*bla*_VIM_ and *bla*_NDM_), vancomycin (*vanA* and *vanB*), and colistin (*mcr* variants), which compromise treatment options for severe infections.^4^^,^^61^ The presence of these genes in surface waters, an accessible environmental compartment, poses a direct risk of human exposure via contaminated water, food, or direct contact, underscoring the environment’s role as a bridge for AMR transmission.

Notably, distinct MDR patterns across environments, such as the high detection of *bla*_CTX-M-1_ in urban parks, moderate levels in suburban lakes and universities, and lower overall burdens in other areas, indicate that specific land uses and human activities (e.g., wastewater discharge, recreational use, and institutional waste) exert unique selective pressures, leading to environment-specific resistance signatures.^62^^,^^63^^,^^64^ This spatial heterogeneity positions urban-associated sites (e.g., university campuses and urban parks) as MDR hotspots, where dense human activity and waste inputs likely drive the accumulation of clinically relevant resistance genes.

The co-occurrence network analysis provides critical insights into the mechanisms sustaining MDR. The strong synergistic relationships between high-prevalence ARGs, such as the near-complete co-occurrence of *gyrA* (*E. coli*) and *parC* (*E. coli*), and frequent co-detection of *ant(2″)-I*, *aac(3)-IV*, and *aph(3′)-III* with *gyrA*, reveal that these genes are not isolated but form integrated resistance cassettes. This integration, coupled with high co-occurrence frequencies, underscores the importance of HGT mechanisms, including conjugation (via plasmid-mediated gene exchange), transformation (uptake of free DNA), and transduction (phage-mediated transfer). These processes are facilitated by MGEs such as plasmids (e.g., IncF and IncI) and integrons (class 1 integrons), which were detected in this study and enable the co-localization and coordinated spread of ARGs (e.g., *bla*_CTX-M_ and *aac(3)-IV*) across diverse bacterial taxa. The dominance of these gene pairs across sites implies shared selective pressures (e.g., antibiotic use and metal co-selection) or efficient HGT mechanisms (e.g., MGEs) that facilitate their persistence, amplifying the risk of resistance dissemination to clinical pathogens.^56^^,^^65^

The severity of these findings lies in the environment’s potential to act as a “reservoir of last resort” for resistance, where co-occurring genes can be transferred to human pathogens, undermining last-line antibiotics. The identification of urban hotspots with high burdens of clinically relevant genes (e.g., carbapenemases and extended-spectrum β-lactamases) heightens public health concerns, as these areas are proximal to human populations and may serve as bridges for transmission.

The risk assessment and preliminary source apportionment in this study provide critical insights into the environmental burden and spatial heterogeneity of ARGs within *E. coli* populations in Huzhou’s surface waters, underscoring the urgency of addressing AMR in this region. The analysis revealed a wide spectrum of risk, with sample risk indices ranging significantly and 25.3% of samples classified as high risk (exceeding the 75th percentile), indicating that a substantial portion of the aquatic environment harbors a heavy load of ARGs. This variability highlights the patchy nature of AMR contamination, where localized hotspots, driven by anthropogenic activities, amplify risk far beyond average levels, demanding targeted surveillance rather than uniform management.

The average ARG detection rate (28.9%) and high-frequency genes (*ant(2″)-I*, 78.2%; *gyrA* and *parC* of *E. coli*, 74.71%; *vanA*, 71.3%) reflect genetic prevalence in *E. coli* isolates. For *vanA* (vancomycin resistance, a last-resort antibiotic), PCR detection in *E. coli* is unexpected and may reflect primer cross-reactivity or rare environmental strains. Its presence does not confirm transferability to human pathogens. The dominance of these genes highlights a genetic burden, but risk assessment requires additional data (e.g., metagenomics and expression analysis). These genes, often co-located with MGEs as implied by their co-occurrence patterns, pose a high risk of HGT to pathogens, potentially eroding the efficacy of life-saving antibiotics. The moderate-to-high frequency of other genes (e.g., *aph(3′)-III* and *aac(3)-IV*) further reflects the broad spectrum of resistance mechanisms circulating in the environment, complicating treatment options for infections.

PCA and clustering revealed the complexity of ARG distribution, with the first five components explaining only 29.2% of variance, indicating that AMR in this system is driven by multiple, interacting factors rather than a single source. The identification of three distinct clusters (high risk, low risk, and moderate-high risk) underscores spatial heterogeneity, as some sites (e.g., urban parks and university campuses) act as hotspots with elevated ARG diversity and abundance, while others (e.g., suburban areas) show lower but non-negligible risk. This clustering provides a framework for prioritizing management, where high-risk clusters (clusters 0 and 2) require immediate intervention to reduce ARG loads, while low-risk clusters (cluster 1) can serve as baselines for monitoring.

These findings derive from a quantitative risk assessment using a risk index (ARG count per sample), detection frequency thresholds (>70% for high-risk genes), and cluster analysis (*k* = 3), which collectively revealed 25.3% of samples as high risk. The presence of last-resort resistance genes (*vanA* and carbapenemases) in surface waters, combined with the high frequency of co-occurring MDR markers, suggests that the environment is a significant amplifier and disseminator of AMR. This not only threatens ecological health but also creates a “reservoir effect,” where resistant *E. coli* and their genes can re-enter human and animal populations via water, food, or direct contact, perpetuating the AMR cycle.

In summary, this study provides a preliminary characterization of ARG prevalence, spatial distribution, and co-occurrence in *E. coli* from Huzhou’s surface waters using targeted PCR. It identifies a significant genetic burden of ARGs, with higher prevalence in urban areas, and suggests potential anthropogenic drivers and HGT hotspots. These findings underscore the need for targeted surveillance and future studies (e.g., metagenomics and sequencing) to validate risks and inform One Health strategies.

### Limitations of the study

This study provides a valuable snapshot of ARGs in *E. coli* from Huzhou’s surface waters but has key limitations. Characterization relied on targeted PCR for a pre-selected set of clinically relevant ARGs, a method with inherent constraints: it does not quantify abundance, expression, or functional resistance, risks cross-reactivity (e.g., with Gram-positive-associated genes like *vanA*/*B*/*X*, *mecC*, *pvl*, or non-target *E. coli* strains with *uidA* positivity), and thus measures only prevalence, not active resistance. While validated primers (from the EU Reference Laboratory for Antimicrobial Resistance (EURL-AR) database) and quality controls (positive/negative controls and optimized annealing temperatures) were used, high detection rates (e.g., *vanA* and *gyrA*) should be interpreted as presumptive ARG presence in *E. coli* (confirmed via *uidA* PCR) rather than absolute activity, and rare non-specific amplification artifacts cannot be fully excluded.

Further, genomic sequencing of isolated *E. coli* strains was not performed, leaving unexplored ARG genetic contexts (e.g., chromosomal vs. plasmid-borne status, association with MGEs like plasmids/integrons, and whether co-occurrence patterns reflect true linkage or PCR artifacts), strain-level epidemiology, and ARG mobility. The investigation also lacked quantification of antibiotic residues in water/sediment (to link environmental selection pressure to ARG abundance) and experimental evaluation of horizontal transfer potential (e.g., via conjugative assays), limiting assessment of HGT risk.

These gaps highlight future needs in adopting metagenomics (to capture the full resistome and ARG context), transcriptomics (to assess expression), and ARG mobility experiments, all aimed at deepening understanding of environmental resistance dynamics.

## Resource availability

### Lead contact

Requests for further information and resources should be directed to and will be fulfilled by the lead contact, Xiang Wang (wangxiang2021@zju.edu.cn).

### Materials availability

Bacterial isolates generated in this study are available from the lead contact upon reasonable request. This study did not generate new, unique reagents.

### Data and code availability

• The antibiotic resistance gene (ARG) presence/absence matrix for all 118 *E. coli* isolates has been included as Table S9 and is publicly available as of the date of publication. Accession codes are listed in the key resources table.
• This article does not report the original code.
• Any additional information required to reanalyze the data reported in this article is available from the lead contact upon request.

## Acknowledgments

This work was funded by the 10.13039/501100001809National Natural Science Foundation of China (82472291 and 82372329), 10.13039/501100004731Zhejiang Provincial Natural Science Foundation of China (LTGD24C010001), and 10.13039/501100009988Huzhou Municipal Science and Technology Bureau (2025YZ33, 2024GZ74, 2024GZ81, and 2021GZ62). We thank all of the people who helped us collect avian fecal samples. We thank all of the people who helped us collect water samples.

## Author contributions

Y.L. and X.W. designed the research. A.W., X.X., W.J., B.L., W.Z., Q.X., L.X., J.S., K.C., Y.G., and Y.Y. performed research. A.W., H.Z., X.Q., and Y.L. performed the bioinformatics analysis. J.H., X.W., H.Z., and Y.L. provided the materials. Y.L., X.W., H.Z., and A.W. wrote the manuscript.

## Declaration of interests

The authors declare no competing interests.

## Declaration of generative AI and AI-assisted technologies in the manuscript preparation process

During the preparation of this work, the authors used DeepSeek-R1 for language refinement. After using this tool, the authors reviewed and edited the content as needed and take full responsibility for the content of the published article.

## STAR★Methods

### Key resources table

**Table:** 

REAGENT or RESOURCE	SOURCE	IDENTIFIER
**Bacterial and virus strains**		

*E. coli* isolates as defined in Tables S1 and S2	This paper	N/A
*E. coli* strain ATCC 25922	ordered from Shanghai Microbial Preservation Co., Ltd	Cat#SHMCC D52291

**Biological samples**		

Fresh water samples (*n*=118) were collected as described in Tables S1 and S2.	This paper; river water, lake, tap-water, stream, and spring water collected from Huzhou, Zhejiang Province, China and outside of Huzhou, China.	N/A

**Chemicals, peptides, and recombinant proteins**		

Lysozyme	Diamond	Cat#A002880
mTEC ChromoSelect Agar	Millipore	Cat#90924
LB Broth Powder, Miller	Sangon Biotech	Cat#A507002
Recombinant Proteinase K (20 mg/ml)	BBI	Cat#B600452
50X TAE (Tris-acetate-EDTA) Electrophoresis Buffer	BBI	Cat#B300033
Agarose, Regular	BBI	Cat#A620014
Taq PCR Master Mix (2X, with Red Dye)	BBI	Cat#B639297
GoldView nucleic acid stains	Yeasen	Cat#10201ES03
pUC19 DNA	BBI	Cat# B610005
DNA Marker (200∼2000 bp)	BBI	Cat#B600339

**Deposited data**		

ARG presence/absence matrix for all 118 *E. coli* isolates together with sample metadata	This paper	Table S9

**Oligonucleotides**		

Primer for *E. coli uidA* gene detection Forward: GGATCTTCCAGAGATATGT TACGTCCTGTAGAAACCCCA	This paper; designed based on known *E. coli uidA* sequence (NCBI Reference Sequence: NC_000913.3)	N/A
Primer for *E. coli uidA* gene detection Reverse: CTGCCGTTCGACGATTCATT GTTTGCCTCCCTGCT	This paper; designed based on known *E. coli uidA* sequence (NCBI Reference Sequence: NC_000913.3)	N/A
Primers for 92 antibiotic resistance genes screening, see Table S8	This paper; National Food Institute, Technical University of Denmark (DTU Food)	Primers were synthesized by Tsingke Biotech. (Beijing, China).

**Software and algorithms**		

Gephi	The Gephi Consortium	Version 0.10.1; https://gephi.org
Python	Python Software Foundation	Version 3.13; https://www.python.org
GraphPad Prism	GraphPad Software	Version 9; https://www.graphpad.com

### Experimental model and study participant details

#### Ethical statement

This research was exclusively limited to the procurement of environmental water specimens. No experimental interventions were carried out on live animals or human participants. Consequently, ethical clearance for animal studies or human research was not necessary. All procedures adhered strictly to local guidelines governing environmental sample collection.

#### Study system and sample allocation

The study focused on *E. coli* isolates obtained from water samples collected in Huzhou City, Zhejiang Province, and other parts of China. These isolates were derived from tap water, rivers, lakes, streams, and mountain springs. A total of 118 freshwater samples were aseptically gathered from different water bodies between January and December 2023. Of these, 89 samples were from Huzhou City and 29 from other regions. Presumptive *E. coli* colonies were isolated using mTEC agar, and subsequently confirmed by *uidA* PCR. For each water sample, all confirmed *E. coli* strains (approximately 10–100 colonies per sample) were retained. To evaluate antibiotic resistance gene (ARG) carriage, genomic DNA from all confirmed isolates of a given sample was pooled, such that each water sample was represented by a single pooled *E. coli* DNA sample for ARG screening.

#### Bacterial strains and culture conditions

*E. coli* strains were initially isolated by centrifuging water samples to concentrate bacteria, then plating on mTEC Agar. Typical colonies were purified on LB agar and confirmed as *E. coli* using PCR targeting the *uidA* gene. All confirmed strains were preserved long-term in glycerol-enriched LB broth at -80°C.

### Method details

#### Study area and site selection

This study was conducted within Huzhou City, located in the northern part of Zhejiang Province, China, a region characterized by its dense network of rivers, lakes, and canals integral to the Taihu Basin. Sampling locations (see Figure 1 for map; Table 1 for specific coordinates) were strategically selected to capture the diversity of surface water bodies within this complex watershed, including major rivers, tributary streams, lakes, and canals. Site selection was driven by the need to assess water quality variations associated with different anthropogenic influences. Consequently, sampling points were positioned both upstream and downstream of major urban rivers to evaluate effluent impacts, adjacent to significant urban centers to gauge urban runoff effects, and within agricultural zones to understand contributions from non-point source pollution. This targeted approach ensured a representative spatial coverage of the key environmental gradients and potential pollution sources affecting Huzhou's aquatic systems.

#### Sampling strategy and collection

The sampling strategy employed seasonal frequency to capture temporal variations, with collections occurring during distinct periods (e.g., winter, spring, summer, fall) over the study's duration. Grab samples were collected at each site using sterile protocols, with a standardized volume (e.g., 1L) to ensure consistency. Strict aseptic techniques were maintained during handling to prevent contamination, including the use of pre-sterilized containers and immediate preservation where required. This approach minimized sample degradation and provided real-time context for water quality conditions influencing the collected samples.

#### Genomic DNA extraction

Genomic DNA was extracted from pure cultures of confirmed *E*. *coli* isolates using established laboratory protocols. Briefly, bacterial cells were harvested from overnight broth cultures via centrifugation. The resulting cell pellet was subjected to lysis using a combination of lysozyme treatment and mechanical disruption (e.g., vortexing), followed by incubation with proteinase K to degrade cellular proteins. Subsequent purification steps involved phenol-chloroform extraction and ethanol precipitation. This process effectively removed proteins, lipids, RNA, and other contaminants. The purified genomic DNA was eluted in 10mM Tris-EDTA (TE) buffer and quantified using NanoDrop, measuring A260/A280 and A260/A230 ratios to assess purity and concentration. DNA integrity was routinely checked via agarose gel electrophoresis.

#### *E*. *coli* isolation and confirmation

Water samples (*n* = 118) were collected from Huzhou City, other regions of Zhejiang Province, and various locations across China. Samples were filtered through membranes using volumes adjusted to expected contamination levels (e.g., 100 mL for surface water). Bacteria retained on the membranes were cultured on selective mTEC agar (modified membrane-Thermotolerant *E. coli* agar) and incubated under conditions promoting thermotolerant coliform growth: 35 ± 0.5°C for 2 hours, followed by 44.5 ± 0.2°C for 22 hours.

Presumptive *E. coli* colonies were identified based on yellow color development—indicative of β-glucuronidase activity—following incubation at 44.5 ± 0.2°C. For confirmation, all presumptive colonies were subjected to PCR targeting the *E. coli*-specific marker gene *uidA* (encoding β-glucuronidase), using the forward primer 5′-GGATCTTCCAGAGATATGTTACGTCCTGTAGAAACCCCA-3′ and reverse primer 5′-CTGCCGTTCGACGATTCATTGTTTGCCTCCCTGCT-3′.^66^

All confirmed *E. coli* strains from each water sample (approximately 10–100 isolates per sample) were retained. To assess antibiotic resistance gene (ARG) carriage, genomic DNA from all confirmed isolates of a given sample was pooled, so that each water sample was represented by a single pooled *E. coli* DNA sample for ARG screening via a targeted PCR panel.

The investigation thus comprised three key steps: (1) isolation of presumptive *E. coli* colonies on mTEC agar; (2) confirmation of isolates via *uidA* PCR; (3) pooling of genomic DNA from confirmed isolates per sample and subsequent ARG screening.

#### Ethical and biosafety statements

This study involved the collection of surface water samples from public aquatic environments in Huzhou, China, with no involvement of human participants, animal subjects, or private data. All sampling activities complied with the Biosafety Law of the People's Republic of China and were approved by the Ethics Committee of Huzhou Normal University (Approval No.: 2023-SGY-06), which confirmed that no ethical review was required for non-invasive environmental sampling.

For biosafety, all *E. coli* isolation, confirmation, and molecular analysis (PCR, DNA extraction) were performed in a Biosafety Level 2 (BSL-2) laboratory at Huzhou University. Standard operating procedures (SOPs) for BSL-2 were followed, including the use of personal protective equipment (lab coats, gloves, eye protection), biological safety cabinets (Class II) for aerosol-generating steps, and autoclaving (121°C, 20 min) of all cultures, consumables, and waste prior to disposal. No genetically modified organisms (GMOs) were used, and all strains were handled to minimize the risk of environmental release.

#### Molecular analysis of ARGs

ARG detection was performed using a targeted polymerase chain reaction (PCR) panel (Figure S12). This approach focused on clinically relevant ARGs representing major antibiotic classes: beta-lactam resistance (*bla*_TEM_, *bla*_CTX-M_, *bla*_CTX-M-1_, *bla*_CTX-M-2_, *bla*_CTX-M-9_, *bla*_ACC-1_, *bla*_DHA-1_, *bla*_FOX_, *bla*_VEB-1_, *bla*_SHV_, *bla*_CMY-1_, *bla*_CMY-2_, *bla*_IMP_, *bla*_SMP_, *bla*_VIM_, *bla*_OXA-48_, *bla*_BIC_, *bla*_KPC_, *bla*_NDM_), chloramphenicol resistance (*cmlA*, *catA1*), colistin resistance (*mcr-1*, *mcr-2*, *mcr-3*, *mcr-4*, *mcr-5*), florphenicol resistance (*floR*), gentamicin resistance (*aac(3)-IV*, *ant(2")-I*, *aac(3)-II*), linezolid resistance (*optrA*), neomycin resistance (*aph(3')-III*, *aph(3')-II*, *aph(3')-I*), quinolone resistance (*gyrA* and *parC* of Salmonella, *gyrA* and *parC* of *E. Coli*, *qnrA*, *qnrB*, *qnrC*, *qnrD*, *qnrS*, *aac(6') Ib-cr*, *qepA*), streptomycin resistance (*strA*, *strB*, *aadA2*, *aadE*), sulfamethoxazole resistance (*sul1*, *sul2*, *sul3*), tetracycline (*tetA*, *tetB*, *tetC*, *tetD*, *tetE*, *tetG*, *tet(H)*, *tet(K)*, *tet(L)*, *tet(M)*, *tet(O)*, *tet(S)*, *tet(T)*, *tet(W)*, *tet(Z)*, *tet(31)*, *tet(32)*, *tet(33)*, *tet(34)*, *tet(39)*), glycopeptides resistance (*vanA*, *vanB*, *vanX*—typically associated with Gram-positive bacteria but sporadically reported in environmental *E. coli*^61^), macrolides resistance (*ermA*, *ermB*, *ermC*, *ermE*, *ermF*), streptogramins resistance (*vat*, *vatB*, *vatD*, *vatE*, *vgaA*, *vgaB*, *vgbA*, *vgbB*), MRSA resistance (*spa*, *mecA*, *mecC*, *pvl*—primarily found in Staphylococcus aureus but with rare *E. coli* isolates reported^29^). The primers used in this study were sourced from Technical University of Denmark, National Food Institute (https://www.food.dtu.dk/english/topics/antimicrobial-resistance/eurl-ar/resources/eurl-ar-primer-list). The primers used in this study were presented in Table S8.

In addition to ARGs, we screened for mobile genetic elements (MGEs) and plasmid markers to assess horizontal gene transfer potential. Plasmid markers included common incompatibility (*inc*) groups (*incF*, *incI*, *incN*, *inc*A/C) and replication initiation genes (*repA*, *repB*).^67^^,^^68^ MGEs targeted were integron class 1 (*intI1*), insertion sequence ISCR1, and transposase gene *tnpA*.^69^^,^^70^ Primers for these markers were sourced from references^68^^,^^69^^,^^70^ and are listed in Table S8. PCR conditions for MGE/plasmid markers were identical to those for ARGs (see below).

PCR (polymerase chain reaction) was performed on a Bio-Rad T100 Thermal Cycler using Taq PCR Master Mix (2X). Temperature profiles for ARG/MGE/plasmid PCR were as follows: initial denaturation at 95°C for 5 min; 35 cycles of denaturation (95°C, 30 s), annealing (55–60°C, 30 s; optimized per primer Tm), and extension (72°C, 1 min per kb); final extension at 72°C for 10 min. Positive controls used *E. coli* strain ATCC 25922 (known to carry *uidA* and select ARGs) and plasmid pUC19 (for MGE/plasmid markers). Negative controls (no-template water) were included in all runs. Primers (sequences in Table S6) were synthesized by Tsingke Biotech. (Beijing, China).

To minimize false positives and non-specific amplification, we implemented: (1) Primers from the validated EURL-AR database (Technical University of Denmark); (2) Positive controls (*E. coli* ATCC 25922, plasmid pUC19) and negative controls (no-template water) in all runs; (3) *uidA* PCR confirmation of *E. coli* before ARG screening; (4) Multiple isolates (average 11 per site/season) to capture intrasite diversity.

#### Data analysis

The comprehensive data analysis employed statistical software (GraphPad Prism 9) to rigorously examine ARG profiles across environmental sites. Initial descriptive statistics quantified ARG prevalence, alongside calculating site-specific multi-drug resistance (MDR) rates. To elucidate spatial patterns, mapping visualized the abundance and distribution of ARGs. The potential environmental and public health risk was evaluated within a structured framework. For exploratory visualization of ARG distribution, principal component analysis (PCA) was applied to binary presence/absence data (detection vs. non-detection) of 92 ARGs—an approach suitable for hypothesis generation but not inferential statistics, given the high correlation and binary nature of the data. To validate spatial heterogeneity, k-means cluster analysis (silhouette coefficient-optimized, *k* = 3) was used as a primary tool for source apportionment. Risk was further assessed via a risk index (number of detected ARGs per sample), detection frequency analysis of critical ARGs, and comparison to World Health Organization (WHO) priority lists.

### Quantification and statistical analysis

Statistical analyses for ARG profiles targeting 92 resistance genes were performed using GraphPad Prism 9. Descriptive statistics were applied to calculate ARG prevalence and site-specific multidrug-resistance rates. Geographic mapping was used to visualize spatial patterns of resistance-gene distribution. Principal component analysis (PCA) was conducted on binary presence-absence matrices of ARGs for exploratory visualization; note that this approach serves for hypothesis generation rather than inferential statistics due to high inter-variable correlation and binary data structure. Silhouette-score-optimized k-means clustering (*k* = 3) was implemented for exploratory source-apportionment analysis. Risk was quantified using three complementary metrics: a sample-level risk index defined as the count of detected ARGs per water sample, detection frequencies of clinically critical ARGs, and comparative profiling referenced against WHO priority pathogen lists. All raw statistical outputs associated with main-text figures, including sample sizes of biological units (independent water samples), FDR-corrected *p* values, silhouette coefficient for k-means clustering, and multivariate analysis outputs, are compiled in Table S10.

### Additional resources

No new websites or forums were created for this study. No supplementary resources were employed in this study.

## Supplemental information

Document S1. Figures S1–S12 and Tables S2–S6

Table S1. Sampling locations and metadata for samples collected in Huzhou

Table S7. Prevalence and distribution of antibiotic resistance genes across sampling sites in Huzhou

Table S8. Primers used for antibiotic resistance gene screening

Table S9. Antibiotic resistance gene presence/absence matrix for all *E. coli* isolates

Table S10. Summary of statistical outputs for Figures 1–6
