# Supplementary material for: High-risk ARGs in environmental E. coli from the Taihu Lake watershed
Source: iScience. 2026 Oct 23;29(11):117986. doi: 10.1016/j.isci.2026.117986 (PMC13720615; doi:10.1016/j.isci.2026.117986)
Supplement: Supplementary file 1 [file mmc1.pdf]

iScience, Volume *29*

Supplemental information

High-risk ARGs in environmental *E. coli* from the Taihu Lake watershed

Afeng Wang, Xinyuan Xu, Weiwei Jiang, Bangyi Li, Wenjie Zhu, Qiuping Xiao, Liu Xu, Junhong Sun, Keyu Chen, Yanfeng Yi, Yingying Guo, Xinhua Qiang, Jie He, Hongchang Zhou, Yibin Lin, and Xiang Wang

Supplementary Table S2. Samples collected from various regions in Zhejiang Province and across China

No.	Sites	Source	Geographical coordinates	Date
SW1	Fenghua, Ningbo,Zhejiang	tap-water	29.663951, 121.413483	16/7/2023
SW2	Xiaoshan, Hangzhou, Zhejiang	tap-water	30.165432, 120.284431	18/7/2023
SW3	Xiaoshan, Hangzhou, Zhejiang	rive water	30.151480, 120.237568	18/7/2023
SW4	Changxing, Huzhou, Zhejiang	rive water	31.001140, 119.921659	19/7/2023
SW5	Deqing, Huzhou, Zhejiang	rive water	30.556407, 119.990067	21/7/2023
SW6	Jinhua, Zhejiang	rive water	29.098447, 119.585770	22/7/2023
SW7	Quzhou, Zhejiang	rive water	28.961861, 118.865822	23/7/2023
SW8	Xia Village, Dongyang, Zhejiang	tap-water	29.361374, 120.389797	26/7/2023
SW9	Xia Village, Dongyang, Zhejiang	spring water	29.365189, 120.389196	26/7/2023
SW10	Xia Village, Dongyang, Zhejiang	stream	29.361972, 120.393145	26/7/2023
SW11	Qinhuai, Nanjing, Jiangsu	tap-water	32.028919, 118.788025	31/7/2023
SW12	Songjiang, Shanghai	rive water	31.028357, 121.206631	29/7/2023
SW13	Jiaxing, Zhejiang	rive water	30.729508, 120.780069	24/7/2023
SW14	Fenghua, Ningbo,Zhejiang	stream	29.702786, 121.336716	25/7/2023
SW15	Ningbo University	rive water	29.906061, 121.640187	25/7/2023
SW16	Chaoyang, Beijing	rive water	39.948467, 116.476892	13/8/2023
SW17	South Railway Station, Beijing	rive water	39.861948, 116.376783	13/8/2023
SW18	Lingang New Area, Shanghai	rive water	30.905568, 121.960670	29/8/2023
SW19	Lingang New Area, Shanghai	tap-water	30.913485, 121.955606	29/8/2023
SW20	Rizhao, Shangdong	seawater	35.357024, 119.556381	12/8/2023
SW21	Qingdao, Shangdong	seawater	36.062311, 120.434601	27/8/2023
SW22	Dinping Town, Linshui, Sichuang	tap-water	30.336873, 106.940128	26/8/2023
SW23	Yanta, Xi'an, Shangxi	rive water	34.144260, 108.901171	25/8/2023
SW24	Puning, Jieyang, Guangdong	spring water	30.890641, 120.131320	27/8/2023
SW25	Weihai, Shangdong	seawater	37.468264, 122.143799	12/8/2023
SW26	Chaozhou, Guangdong	seawater	23.547244, 117.081505	12/8/2023

SW27	Dongtou, Wenzhou, Zhejiang	seawater	27.837801, 121.179405	12/8/2023
SW28	Huludao, Liaoning	seawater	40.699173, 120.947937	11/7/2023
SW29	Zhangzhou, Fujian	rive water	24.438922, 117.820529	19/8/2023
SW30	Zhangzhou, Fujian	rive water	24.441188, 117.810272	19/8/2023
SW31	Zhangzhou, Fujian	rive water	24.452916, 117.816233	19/8/2023

Supplementary Table S3. Detection rates of antibiotic resistance genes (ARGs) in water samples from Huzhou

Samples	Detection rate (%)
HZ01	31.52
HZ02	29.34
HZ03	29.34
HZ04	15.21
HZ05	31.52
HZ06	26.08
HZ07	29.34
HZ08	25.00
HZ09	34.78
HZ10	35.86
HZ11	29.34
HZ12	27.17
HZ13	36.95
HZ14	28.26
HZ15	30.43
HZ16	51.08
HZ17	20.65
HZ18	50.00
HZ19	41.30
HZ20	29.34
HZ21	26.08
HZ22	23.91
HZ23	28.26
HZ24	42.39
HZ25	30.43
HZ26	36.95
HZ27	61.95
HZ28	38.04
HZ29	40.21
HZ30	40.21
HZ31	38.04
HZ32	28.26
HZ33	31.52
HZ34	28.26
HZ35	21.73
HZ36	17.39
HZ37	19.56
HZ38	28.26
HZ39	41.30
HZ40	27.17
HZ41	30.43
HZ42	28.26

HZ43	29.34
HZ44	33.69
HZ45	29.34
HZ46	26.08
HZ47	40.21
HZ48	32.60
HZ49	18.47
HZ50	38.04
HZ51	28.26
HZ52	27.17
HZ53	39.13
HZ54	25.00
HZ55	27.17
HZ56	15.21
HZ57	32.60
HZ58	28.26
HZ59	4.347
HZ60	27.17
HZ61	10.86
HZ62	27.17
HZ63	29.347
HZ64	22.82
HZ65	18.47
HZ66	19.56
HZ67	26.08
HZ68	17.39
HZ69	17.39
HZ70	19.56
HZ71	9.78
HZ72	15.21
HZ73	11.95
HZ74	19.56
HZ75	23.91
HZ76	8.69
HZ77	19.56
HZ78	16.30
HZ79	39.13
HZ80	34.78
HZ81	35.86
HZ82	44.565
HZ83	44.56
HZ84	27.17
HZ85	33.69
HZ86	35.86
HZ87	38.04

Supplementary Table S4. Detection rates of antibiotic resistance genes (ARGs) in water samples from non-Huzhou

Samples	Detection rate (%)
SW1	32.61
SW2	28.26
SW3	21.74
SW4	18.48
SW5	0
SW6	35.87
SW7	3.26
SW8	27.17
SW9	17.39
SW10	16.3
SW11	0
SW12	18.48
SW13	34.78
SW14	28.26
SW15	28.26
SW16	0
SW17	19.57
SW18	3.26
SW19	17.39
SW20	28.26
SW21	22.83
SW22	8.7
SW23	0
SW24	20.65
SW25	19.57
SW26	19.57
SW27	27.17
SW28	11.96
SW29	17.39
SW30	9.78
SW31	5.43

Supplementary Table S5. Comparison of detection rates and statistical significance of targeted antibiotic resistance genes between Huzhou and non-Huzhou samples

Class	Resistance Gene	Huzhou Detection Rate	Non-Huzhou Detection Rate	Detection Rate Difference	Chi-square Statistic	*P*-value	Significance
Betalactams	bla_{TEM}	0.1839	0.0000	0.1839	5.1197	0.1170	Not Significant
	$bla_{CTX\text{-}M}$	0.5632	0.1290	0.4342	15.7044	0.0033	Significant
	$bla_{CTX\text{-}M\text{-}1}$	0.6092	0.2258	0.3834	11.9517	0.0162	Significant
	$bla_{CTX\text{-}M\text{-}2}$	0.0000	0.0000	0.0000	ND	ND	ND
	$bla_{CTX\text{-}M9}$	0.6437	0.3548	0.2888	6.6378	0.0808	Not Significant
	$bla_{ACC\text{-}1}$	0.2759	0.0645	0.2113	4.7762	0.1223	Not Significant
	$bla_{DHA\text{-}1}$	0.0000	0.0000	0.0000	ND	ND	ND
	bla_{FOX}	0.5172	0.3226	0.1947	2.7420	0.3026	Not Significant
	$bla_{VEB\text{-}1}$	0.0805	0.0323	0.0482	0.2506	0.8315	Not Significant
	bla_{SHV}	0.2644	0.1290	0.1353	1.6674	0.4375	Not Significant
	$bla_{CM1\text{-}1}$	0.5747	0.3226	0.2521	4.8485	0.1223	Not Significant
	$bla_{CM1\text{-}2}$	0.5402	0.2258	0.3144	7.8810	0.0494	Significant
	bla_{IMP}	0.0230	0.0000	0.0230	0.0017	1.0000	Not Significant
	bla_{SMP}	0.4828	0.2258	0.2570	5.2016	0.1170	Not Significant
	bla_{VIM}	0.6667	0.3871	0.2796	6.2897	0.0864	Not Significant
	$bla_{OXA\text{-}48}$	0.0690	0.0000	0.0690	1.0501	0.5549	Not Significant
	bla_{BIC}	0.2874	0.2258	0.0615	0.1820	0.8764	Not Significant
	bla_{KPC}	0.0805	0.0000	0.0805	1.4057	0.4562	Not Significant
	bla_{NDM}	0.2529	0.2581	-0.0052	0.0000	1.0000	Not Significant
Chloramphenicol	*cmlA*	0.6552	0.4516	0.2036	3.1480	0.2706	Not Significant
	catA1	0.0920	0.0000	0.0920	1.7760	0.4168	Not Significant
Colistin	*mcr-1*	0.1149	0.0968	0.0182	0.0000	1.0000	Not Significant
	mcr-2	0.0115	0.0645	-0.0530	0.8948	0.6126	Not Significant
	mcr-3	0.0345	0.0000	0.0345	0.1466	0.8923	Not Significant
	mcr-4	0.0115	0.0323	-0.0208	0.0000	1.0000	Not Significant
	mcr-5	0.0460	0.0323	0.0137	0.0000	1.0000	Not Significant
Florphenicol	*floR*	0.1839	0.1613	0.0226	0.0001	1.0000	Not Significant
Gentamicin	*aac(3)-IV*	0.6897	0.6129	0.0768	0.3111	0.8024	Not Significant
	ant(2")-I	0.7816	0.6452	0.1364	1.5824	0.4524	Not Significant
	aac(3)-II	0.2759	0.0645	0.2113	4.7762	0.1223	Not Significant
Linezolid	*optrA*	0.0920	0.0968	-0.0048	0.0000	1.0000	Not Significant
Neomycin	*aph(3')-III*	0.7011	0.7097	-0.0085	0.0000	1.0000	Not Significant
	aph(3')-II	0.3103	0.0968	0.2136	4.4297	0.1429	Not Significant
	aph(3')-I	0.0575	0.0968	-0.0393	0.1098	0.9151	Not Significant
Quinolones	*g1rA* Salmonella	0.5747	0.2258	0.3489	9.7889	0.0260	Significant
	parC Salmonella	0.5402	0.2258	0.3144	7.8810	0.0494	Significant
	gyrA E. Coli	0.7471	0.6129	0.1342	1.4065	0.4562	Not Significant
	parC E. Coli	0.7471	0.6129	0.1342	1.4065	0.4562	Not Significant

	qnrA	0.0805	0.0000	0.0805	1.4057	0.4562	Not Significant
	qnrB	0.0920	0.0645	0.0274	0.0091	1.0000	Not Significant
	qnrC	0.0115	0.0000	0.0115	0.0000	1.0000	Not Significant
	qnrD	0.4943	0.3871	0.1072	0.6680	0.6819	Not Significant
	qnrS	0.1609	0.0968	0.0641	0.3312	0.8024	Not Significant
	aac(6') Ib-cr	0.2414	0.0968	0.1446	2.1248	0.3583	Not Significant
	qepA	0.1609	0.0323	0.1287	2.3489	0.3188	Not Significant
Streptomycin	*strA*	0.4023	0.4839	-0.0816	0.3336	0.8024	Not Significant
	strB	0.4483	0.5484	-0.1001	0.5610	0.7344	Not Significant
	aadA2	0.4828	0.0323	0.4505	18.1295	0.0018	Significant
	aadE	0.1494	0.0968	0.0527	0.1847	0.8764	Not Significant
Sulfamethoxazole	*sul1*	0.4598	0.4839	-0.0241	0.0005	1.0000	Not Significant
	sul2	0.6207	0.2581	0.3626	10.6427	0.0219	Significant
	sul3	0.1494	0.0645	0.0849	0.8184	0.6381	Not Significant
Tetracycline	*tetA*	0.5172	0.1613	0.3560	10.4465	0.0219	Significant
	tetB	0.0345	0.0000	0.0345	0.1466	0.8923	Not Significant
	tetC	0.3333	0.5161	-0.1828	2.5087	0.3045	Not Significant
	tetD	0.2529	0.0968	0.1561	2.4660	0.3045	Not Significant
	tetE	0.3333	0.1290	0.2043	3.7757	0.1928	Not Significant
	tetG	0.0575	0.0968	-0.0393	0.1098	0.9151	Not Significant
	tet (H)	0.0920	0.0000	0.0920	1.7760	0.4168	Not Significant
	tet(K)	0.1264	0.0645	0.0619	0.3739	0.8023	Not Significant
	tet(L)	0.0805	0.0645	0.0159	0.0000	1.0000	Not Significant
	tet(M)	0.0575	0.0000	0.0575	0.7137	0.6687	Not Significant
	tet(O)	0.5402	0.2581	0.2822	6.2226	0.0864	Not Significant
	tet(S)	0.6437	0.3871	0.2566	5.1562	0.1170	Not Significant
	tet(T)	0.0920	0.1613	-0.0693	0.5252	0.7448	Not Significant
	tet(W)	0.1264	0.0000	0.1264	2.9561	0.2820	Not Significant
	tet(Z)	0.5287	0.2581	0.2707	5.7000	0.1079	Not Significant
	tet(31)	0.0230	0.0000	0.0230	0.0017	1.0000	Not Significant
	tet(32)	0.4713	0.1613	0.3100	7.9754	0.0494	Significant
	tet(33)	0.0230	0.0645	-0.0415	0.2695	0.8265	Not Significant
	tet(34)	0.1724	0.0323	0.1402	2.7281	0.3026	Not Significant
	tet(39)	0.0345	0.0645	-0.0300	0.0375	1.0000	Not Significant
Glycopeptides	*vanA*	0.7126	0.5806	0.1320	1.2695	0.4845	Not Significant
	vanB	0.3793	0.1290	0.2503	5.5396	0.1103	Not Significant
	van X	0.0920	0.0000	0.0920	1.7760	0.4168	Not Significant
Macrolides	*ermA*	0.0575	0.0000	0.0575	0.7137	0.6687	Not Significant
	ermB	0.3103	0.3871	-0.0768	0.3111	0.8024	Not Significant
	ermC	0.2989	0.1290	0.1698	2.6384	0.3045	Not Significant
	ermE	0.5747	0.3871	0.1876	2.5179	0.3045	Not Significant
	ermF	0.0345	0.0323	0.0022	0.0000	1.0000	Not Significant
Streptogramins	*vat*	0.3218	0.1935	0.1283	1.2618	0.4845	Not Significant
	vatB	0.0345	0.0323	0.0022	0.0000	1.0000	Not Significant
	vatD	0.1264	0.0645	0.0619	0.3739	0.8023	Not Significant
	vatE	0.1494	0.0000	0.1494	3.7930	0.1928	Not Significant

	vgaA	0.2759	0.1935	0.0823	0.4403	0.7779	Not Significant
	vgaB	0.4598	0.2581	0.2017	3.0630	0.2742	Not Significant
	vgbA	0.2069	0.1290	0.0779	0.4723	0.7681	Not Significant
	vgbB	0.1149	0.0000	0.1149	2.5523	0.3045	Not Significant
	spa	0.6322	0.4839	0.1483	1.5142	0.4562	Not Significant
MRSA	*mecA*	0.0115	0.0000	0.0115	0.0000	1.0000	Not Significant
	mecC	0.4828	0.1935	0.2892	6.7691	0.0808	Not Significant
	pvl	0.0000	0.0000	0.0000	ND	ND	ND

Supplementary Table S6. Summary of detection rates for different ARG categories in non-Huzhou

Rank	Category	Number of Genes	Average Detection Rate (%)	Minimum Detection Rate (%)	Maximum Detection Rate (%)
1	Gentamicin	3	44.09	0.00	66.67
2	Sulfamethoxazole	3	26.89	0.00	66.67
3	Glycopeptides	3	23.66	0.00	66.67
4	Chloramphenicol	2	22.58	0.00	50.00
5	Streptomycin	4	29.03	0.00	75.00
6	Neomycin	3	30.11	0.00	66.67
7	Quinolones	11	21.41	0.00	45.45
8	β-lactams	19	15.28	0.00	42.11
9	MRSA	4	50.00	0.00	50.00
10	Macrolides	5	18.71	0.00	60.00
11	Tetracycline	20	12.58	0.00	45.00
12	Streptogramins	8	10.89	0.00	37.50
13	Florfenicol	1	16.13	0.00	100.00
14	Linezolid	1	9.38	0.00	100.00
15	Colistin	5	4.52	0.00	40.00

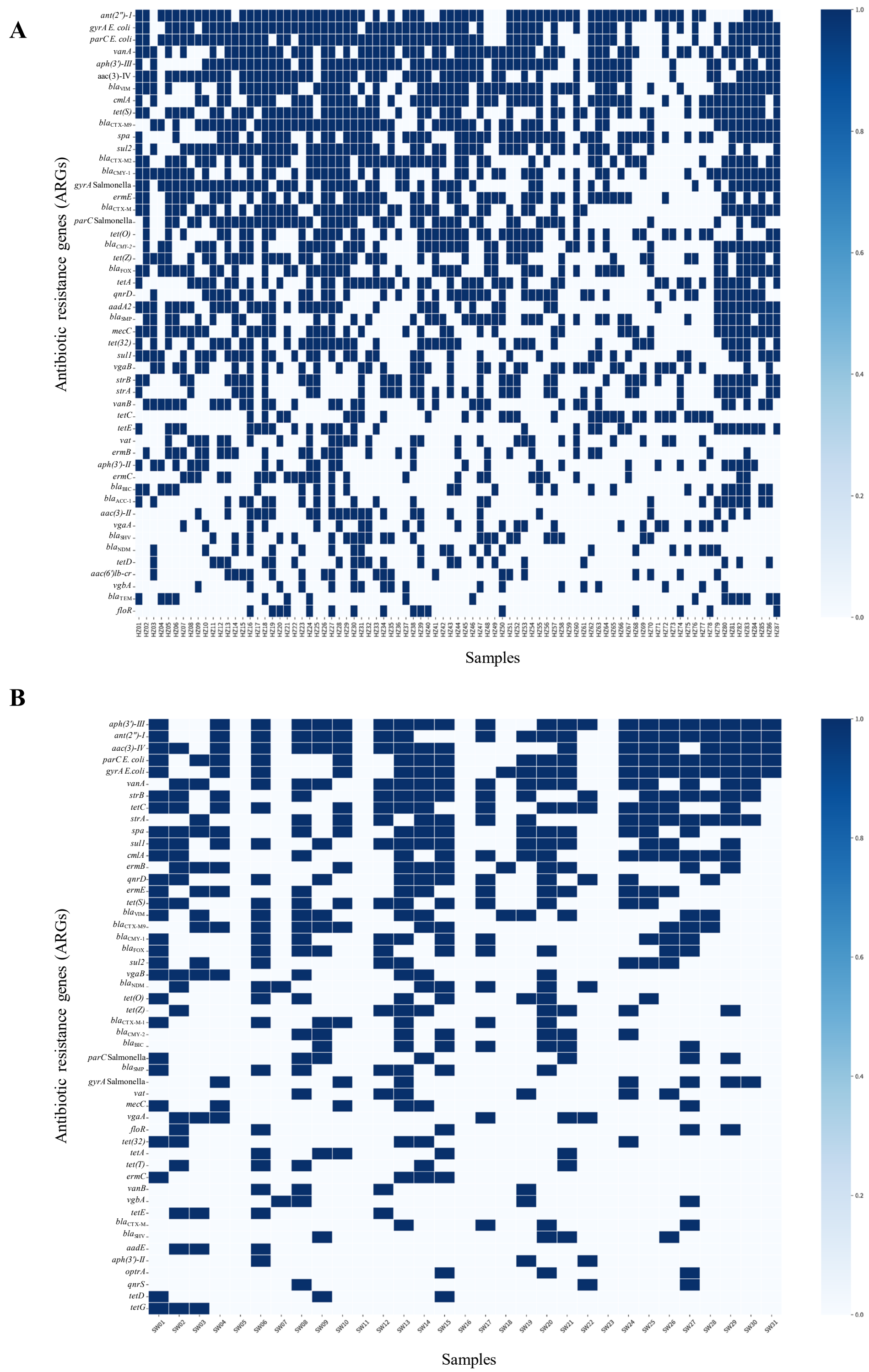

Supplementary Figure S1. Detection of ARGs across different regions. The heatmap indicates the presence (blue) or absence (light color) of ARGs in samples collected from the Huzhou region (A) and from non-Huzhou regions (B).

A

B

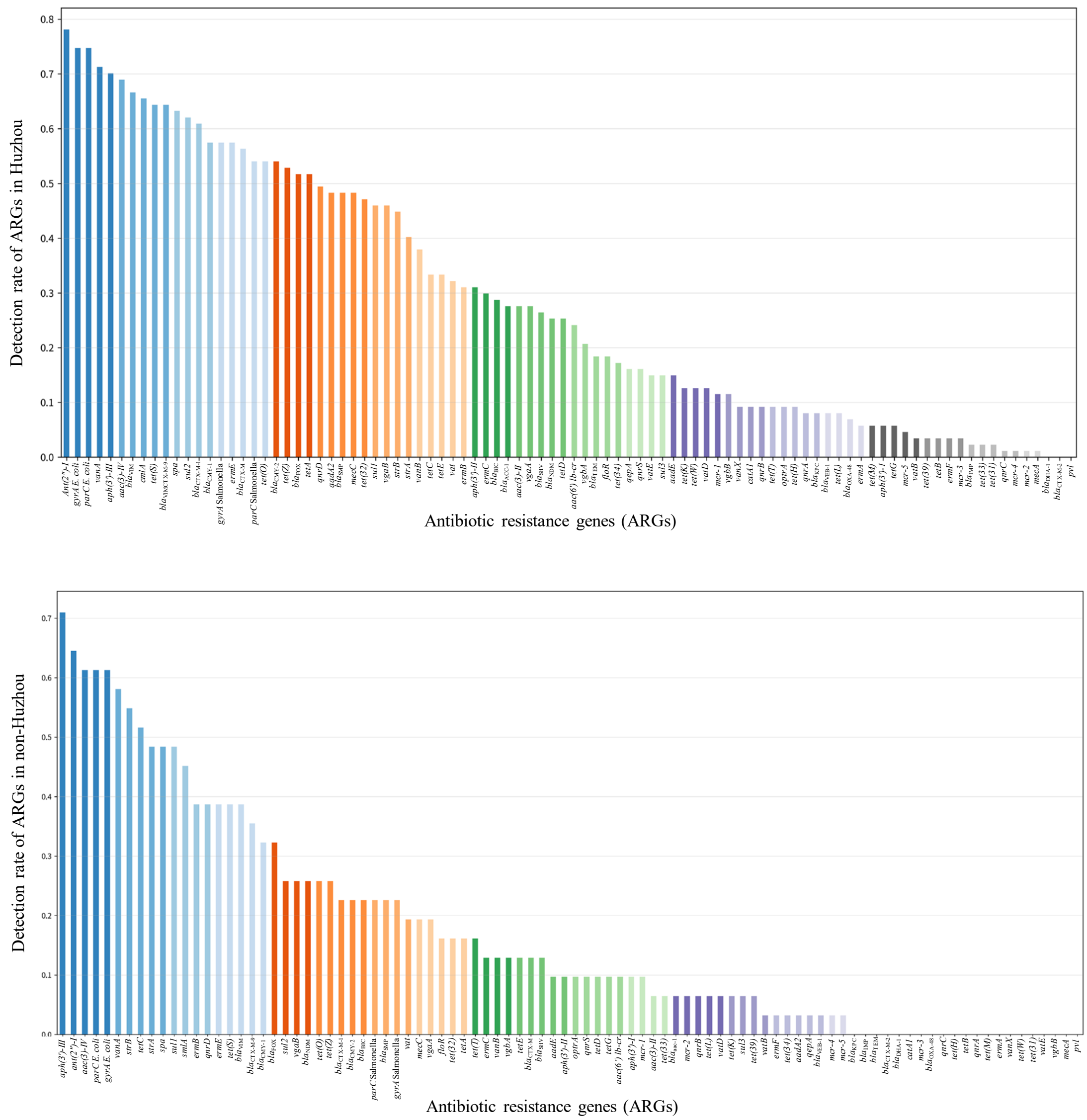

Supplementary Figure S2. Comparative analysis of ARG prevalence between Huzhou and non-Huzhou Areas. This figure presents a side-by-side comparison of the detection rates for a comprehensive panel of ARGs across two distinct geographical regions, displayed as (A) Huzhou and (B) non-Huzhou. The x-axis enumerates various individual resistance genes, and the y-axis quantifies their detection rates. A color gradient is applied across the bars within each panel to visually distinguish between the genes, facilitating a clear comparison of their relative prevalence within and between the two regions.

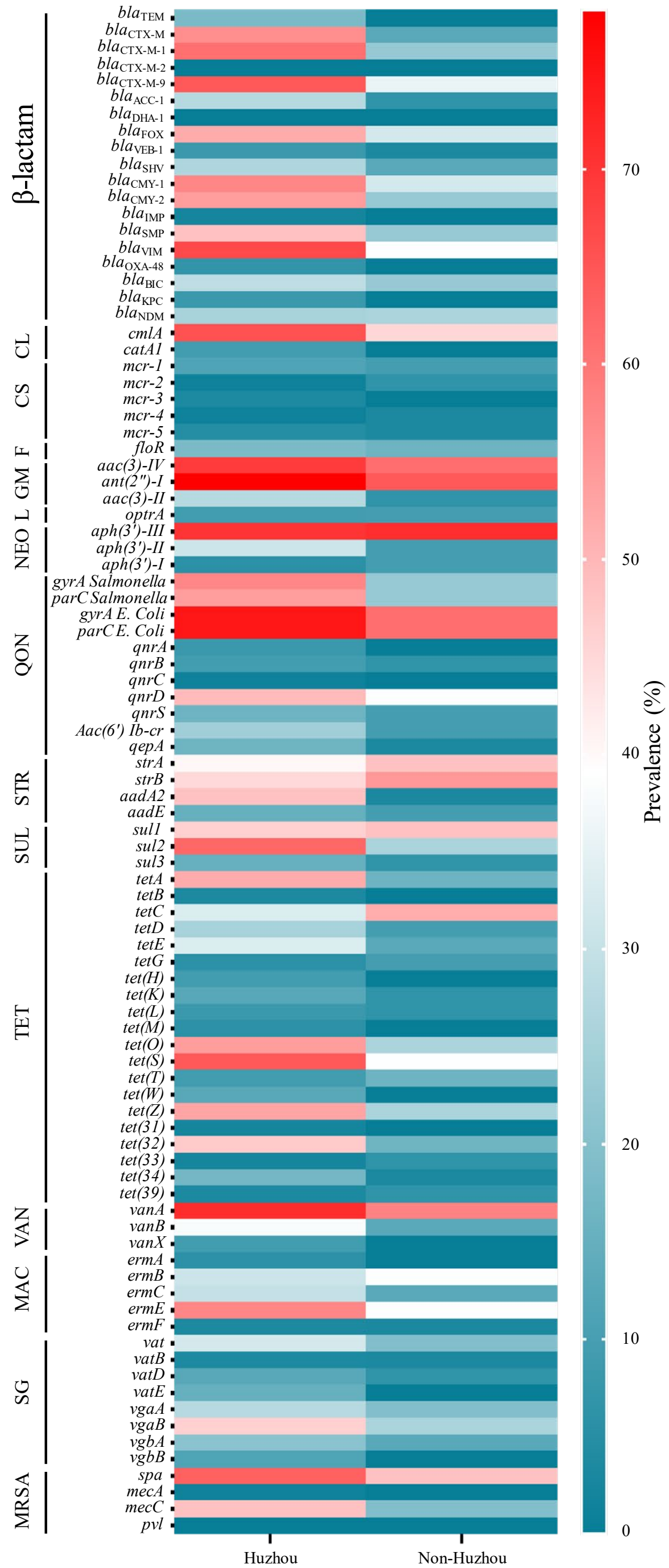

Supplementary Figure S3. Comparative heatmap of ARG detection rates between Huzhou and Non-Huzhou regions. This heatmap illustrates the detection rates of 92 ARGs, categorized by class, comparing samples from the Huzhou region versus non-Huzhou regions. The vertical axis lists all tested resistance genes along with their classifications. The horizontal axis represents the two sample groups: "Huzhou" and "Non-Huzhou". A color gradient from blue to red corresponds to detection rates ranging from 0% to 70%, as shown in the adjacent scale.

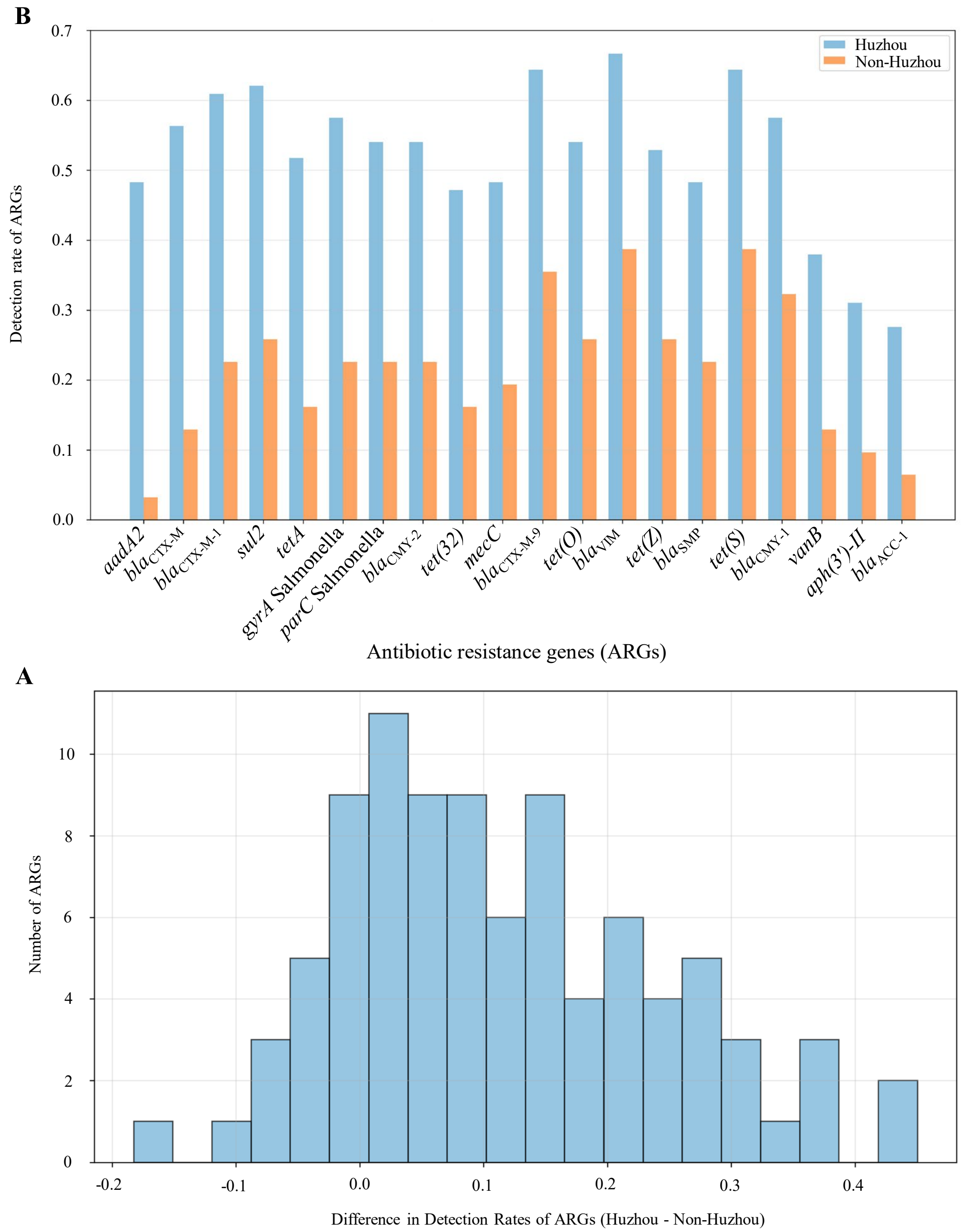

Supplementary Figure S4. A comparative analysis of ARG prevalence between the Huzhou region and non-Huzhou areas. (A) Top 20 ARGs with the largest detection rate differences. The bar chart displays the specific detection rates for 20 key resistance genes, such as *aadA2*, CTX-M1 group, and *tetA*, with paired blue and orange bars representing Huzhou and non-Huzhou, respectively, revealing notable regional variations for most genes. (B) ARG detection rate difference distribution. The bar chart illustrates the frequency distribution of the detection rate differences (calculated as Huzhou minus non-Huzhou) across all genes, with the x-axis showing difference values from -0.2 to 0.4 and the y-axis indicating gene counts, highlighting that most differences cluster near zero.

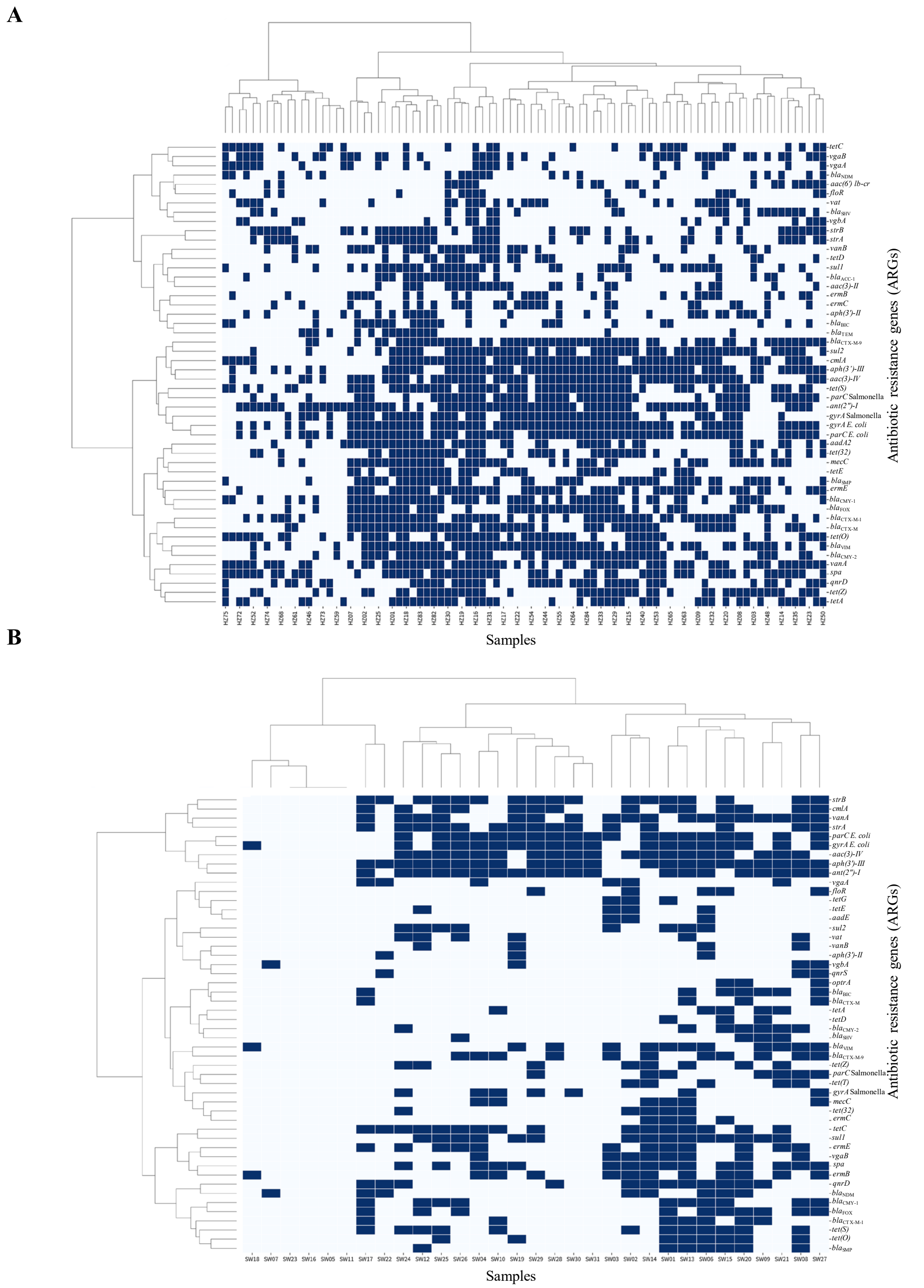

Supplementary Figure S5. Cluster analysis of ARG detection patterns across two regions. (A) Heatmap depicting the presence (blue) or absence (light color) of the top 50 most frequently detected ARGs in samples from the Huzhou region. (B) Heatmap illustrating the detection patterns of the top 50 ARGs in non-Huzhou regions. Clustering was performed based on the co-occurrence of resistance genes across samples as well as the similarity in detection profiles among different genes.

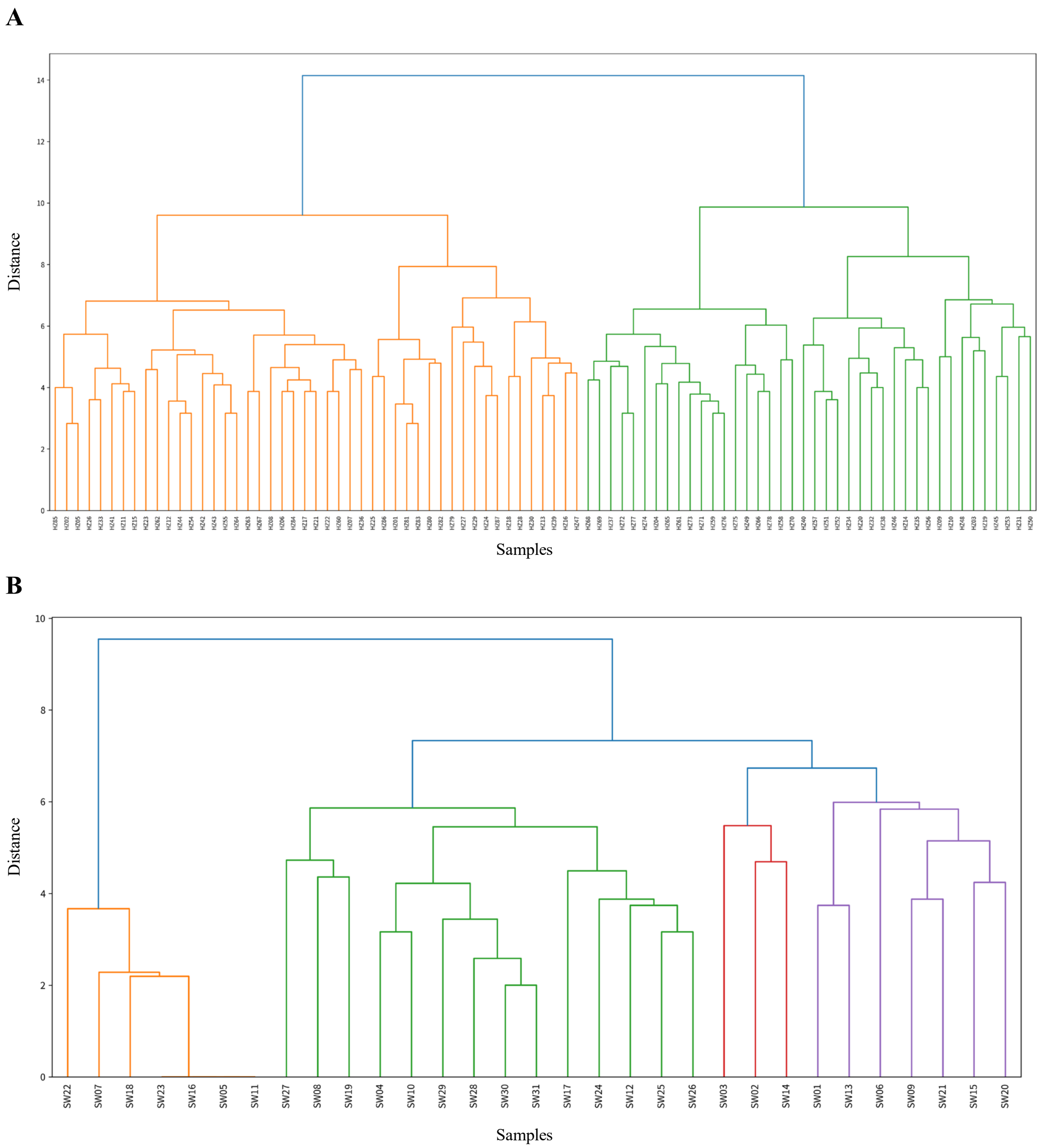

Supplementary Figure S6. Hierarchical clustering analysis of ARG detection rates in samples from Huzhou and Non-Huzhou regions. This figure presents a comparative hierarchical clustering analysis of ARG detection profiles across two distinct geographical sample sets. (A) The clustering results for 87 samples from the Huzhou region (HZ01 to HZ87), revealing a primary bifurcation into two major clusters: a larger group of 47 samples (in orange) and a smaller group of 40 samples (in green), separated by a high dissimilarity level. This suggests a notable dichotomy in ARG carriage patterns within the Huzhou samples. (B) The analysis for 31 samples from non-Huzhou regions (S01 to S31), which exhibits a more complex branching structure with multiple distinct subgroups.

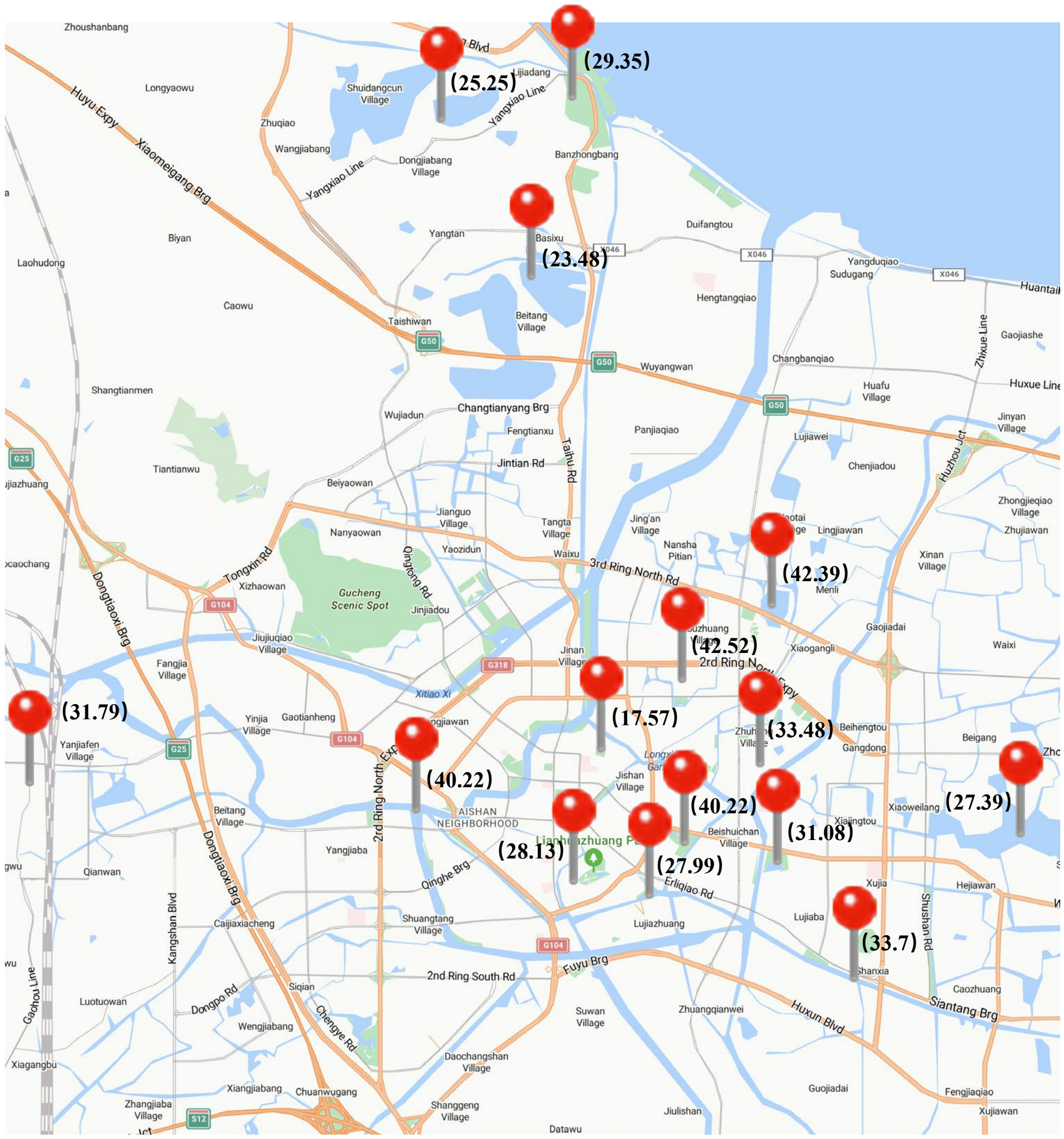

Supplementary Figure S7. Spatial patterns of ARG distribution. Map showing the geographic locations of sampling points (n=15) within the Huzhou region, China. Each point represents a water sampling site where *Escherichia coli* was isolated. The spatial distribution illustrates the detected ARGs across these sites. The pattern suggests heterogeneity in ARG prevalence throughout the study area.

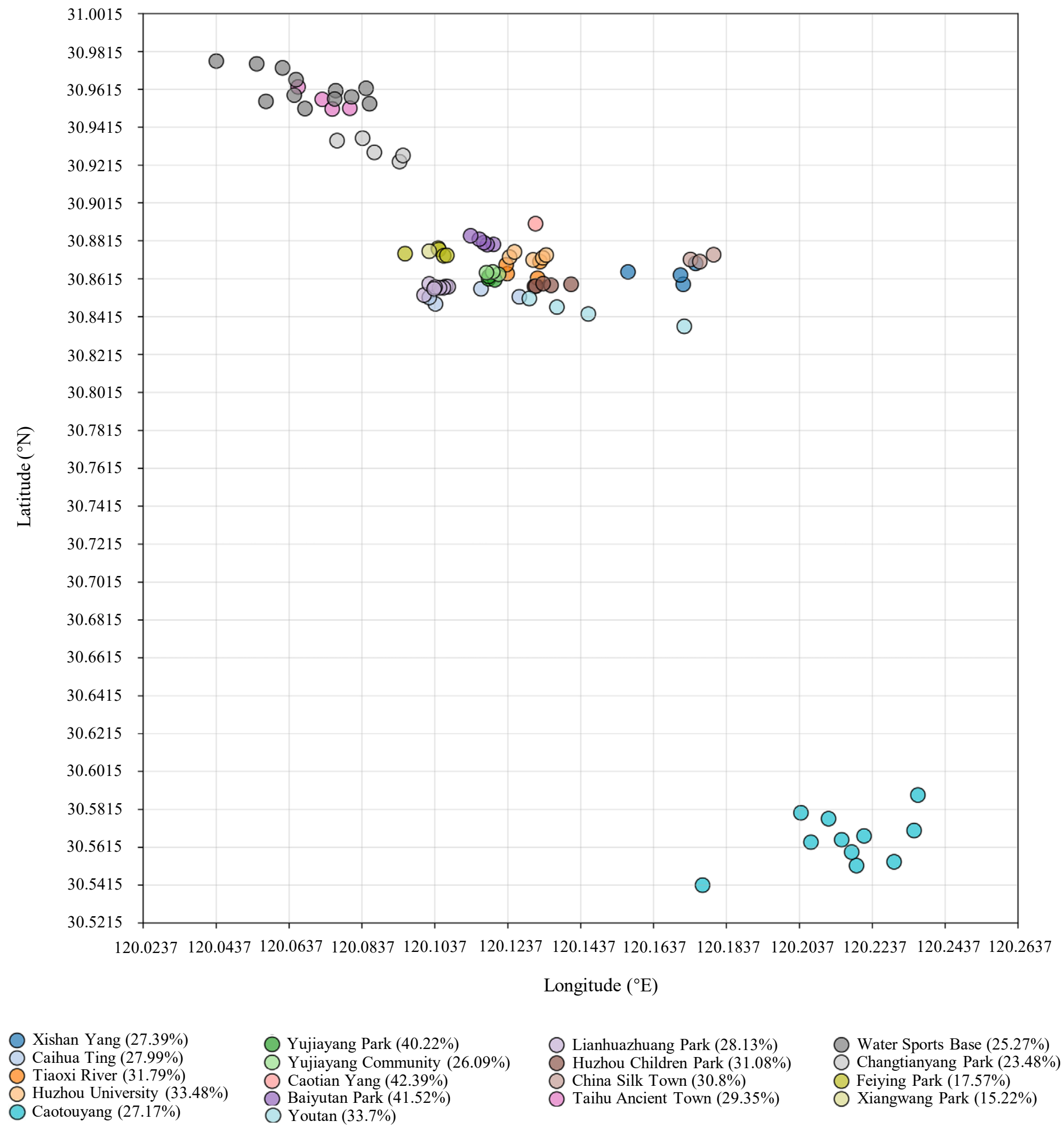

Supplementary Figure S8. Geospatial distribution of environmental sampling sites across Huzhou City, Zhejiang Province, China. The figure presents a geospatial scatter plot illustrating the sampling locations within Huzhou City, Zhejiang Province, China. Various sampling sites are distinctly marked by colored dots, each corresponding to a specific location as defined in the legend. These sites encompass a diverse range of urban and natural environments, including specific water bodies like Xishan Yang, TiaoXi River, Caotianyang, Caotouyang, and Youtan; educational and institutional zones such as Huzhou University; public parks and recreational areas like Yujiayang Park, Baiyutan Park, Lianhuazhuang Park, Huzhou Children Park, Feiying Park, and Xiangwang Park; community spaces represented by Yujiayang Community; cultural and commercial hubs including China Silk Town and Taihu Ancient Town; a specialized Water Sports Base; and a natural reserve, Changtianyang Wetland Park. An additional point is labeled Caihua Ting. The detection rates of ARGs at different sampling points are also displayed.

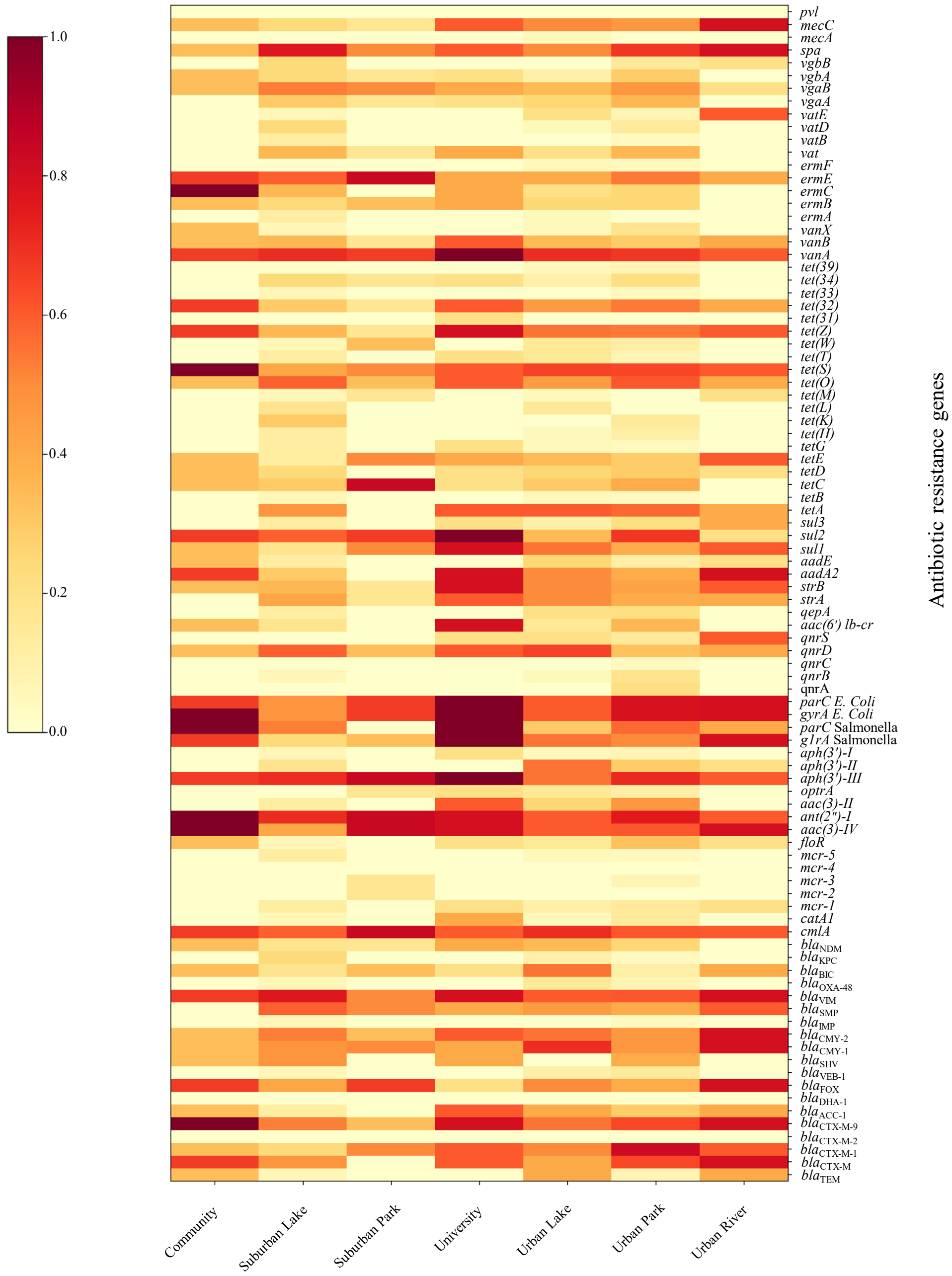

Supplementary Figure S9. Environmental reservoirs of antibiotic resistance reveal gene prevalence across habitats in heatmap analysis. This heatmap illustrates the detection rates of key antibiotic resistance genes across seven distinct environmental habitats, ranging from urban and suburban aquatic systems to public and institutional spaces. Each row corresponds to a specific resistance gene, while each column represents an environmental category including Urban River, Urban Park, Urban Lake, University, Suburban Park, Suburban Lake, and Community samples. Color intensity within each cell, scaled from 0.0 (pale yellow) to 1.0 (deep red), denotes the relative abundance or detection frequency of a given gene in the corresponding environment.

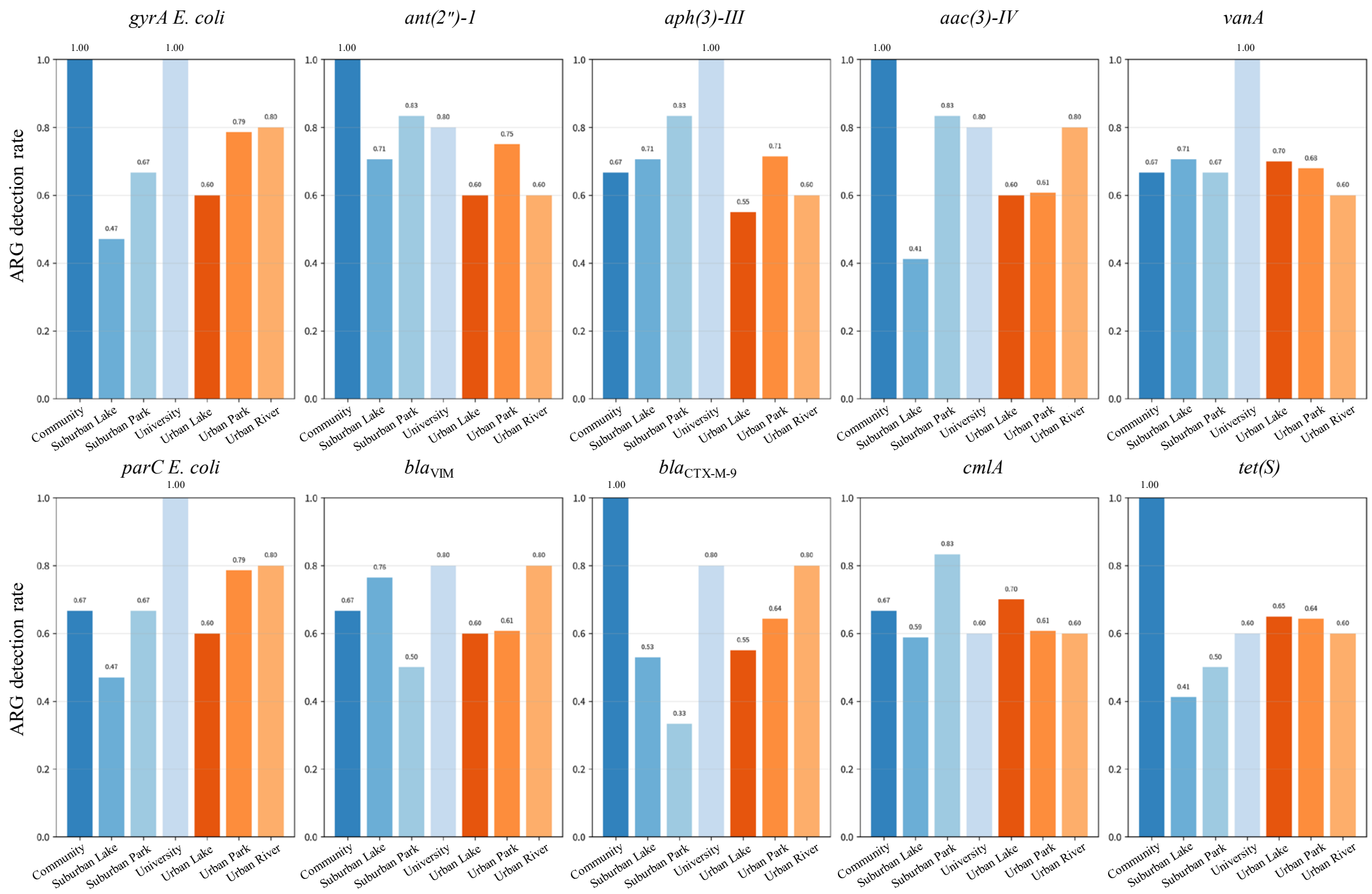

Supplementary Figure S10. Regional distribution of the Top 10 ARGs. This figure illustrates the comparative detection rates of the top 10 antibiotic-resistance genes across seven distinct regional settings: Community, Suburban Lake, Suburban Park, University, Urban Lake, Urban Park, and Urban River. Each of the ten bar charts corresponds to a specific resistance gene, including *gyrA E. coli*, *ant*(2")-I, *aph*(3")-III, and others, with the x-axis denoting the regions and the y-axis representing the detection rate scaled from 0 to 1.

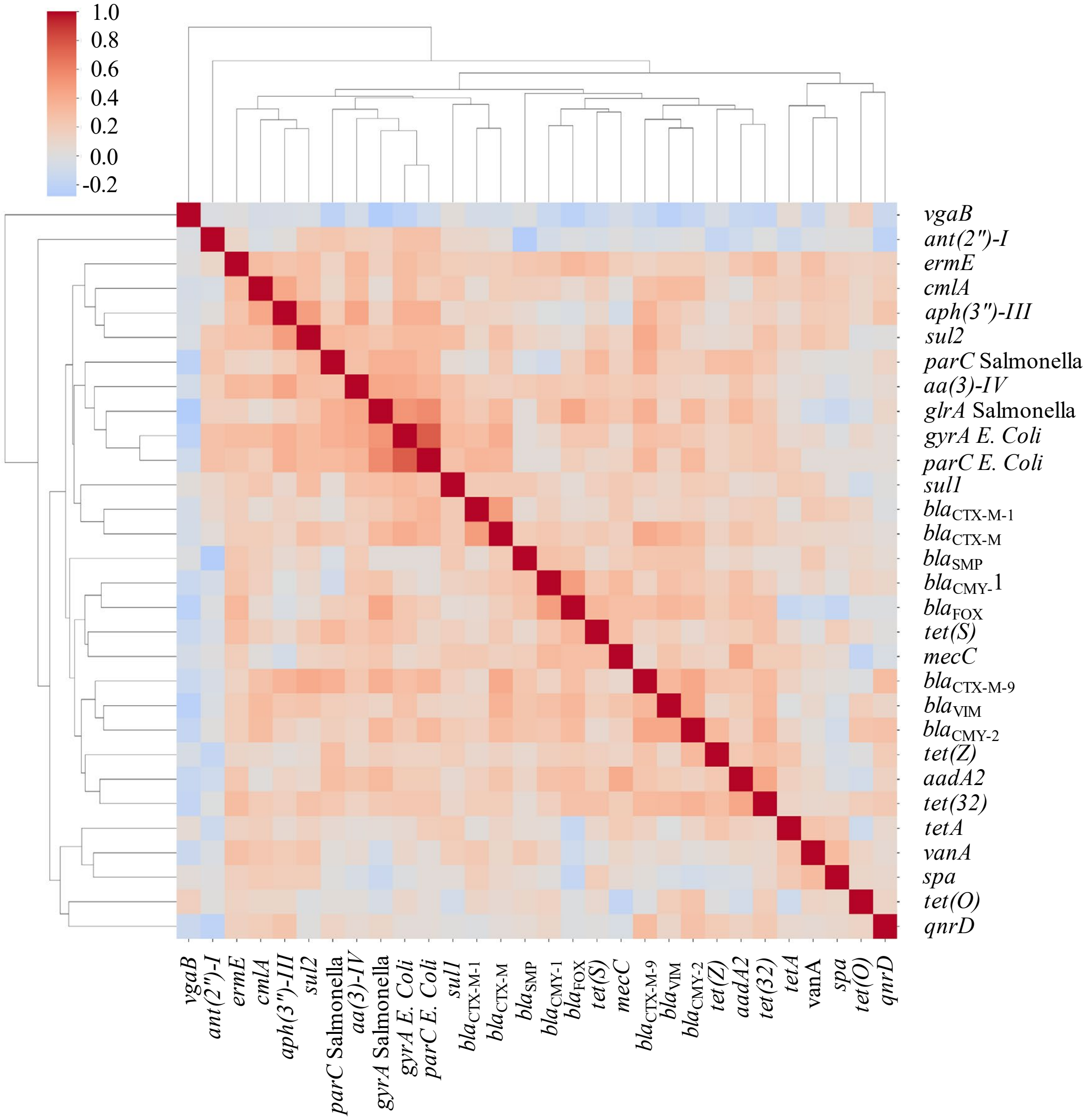

Supplementary Figure S11. Full co-occurrence matrix heatmap of high-frequency ARGs. Heatmap showing both positive and negative correlations (r ranges from -0.2 to 1.0) between high-frequency ARGs, highlighting synergistic and antagonistic co-occurrence patterns.

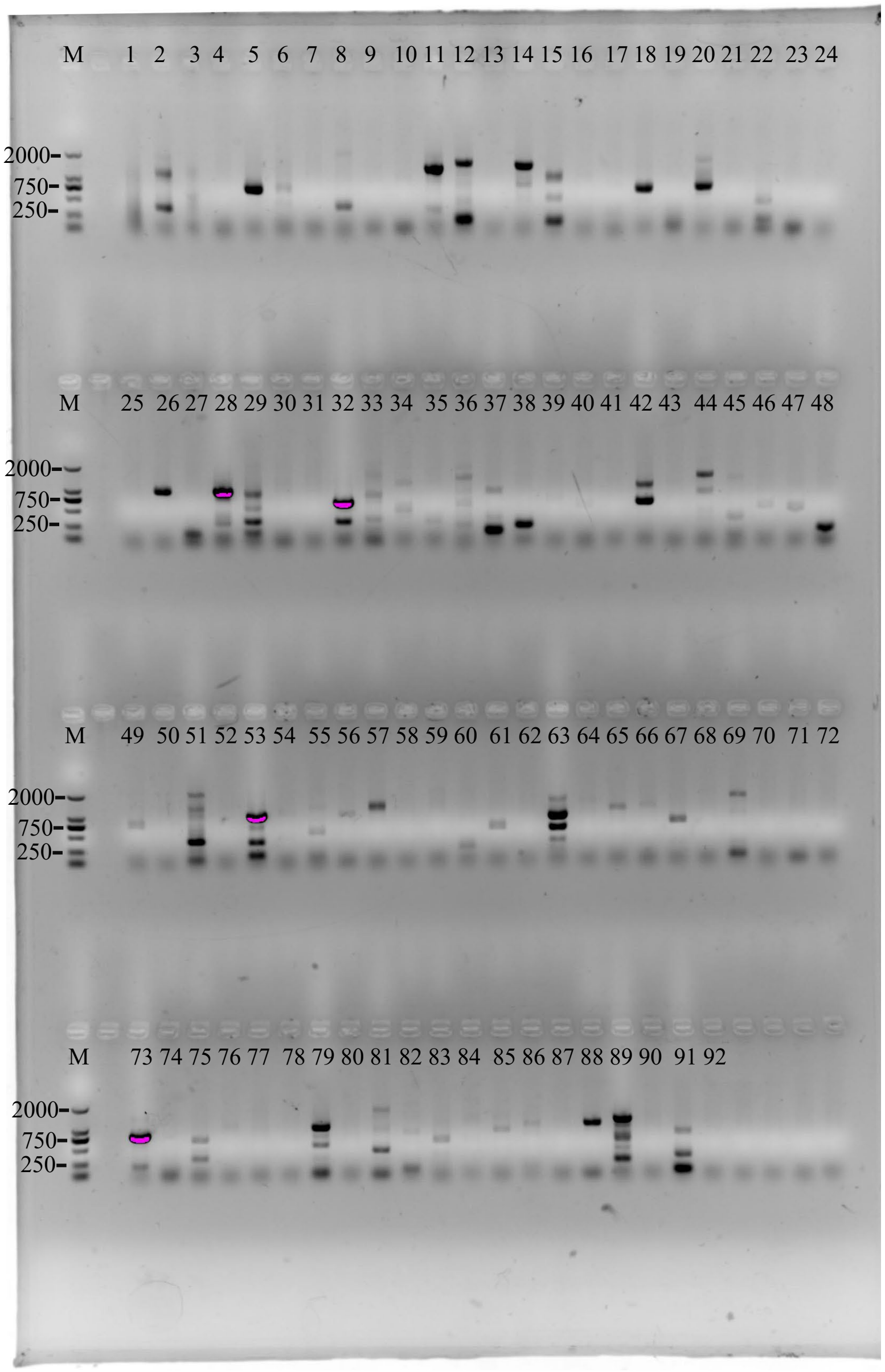

Supplementary Figure S12. PCR-based antibiotics resistance gene fingerprint patterns of *E. coli* strain isolated from HZ53 sample. 1: *bla*TEM;2-5: *bla*CTX group; 6-10: *bla*ACC, *bla*DHA, *bla*FOX, *bla*VEB, *bla*SHV groups; 11-12: *bla*CMY group; 13-19: *bla*IMP, *bla*SMP, *bla*VIM, *bla*OXA, *bla*BIC, *bla*KPC, *bla*NDM groups; 20-21: chloramphenicol *cmlA* and *catA1*; 22-26: colistin *mcr1-5*; 27: florphenicol *floR*; 28-30: gentamicin aac(3)-IV, ant(2″)-I, aac(3)-II; 31: linezolid *optrA*; 32-34: neomycin *aph(3')-III-I*; 35-45: quinolones *gyrA*, *parC*, *gyrA*, *parC*, *qnrA-D*, *qnrS, aac(6')bl-cr*, *qepA*; 46-49: streptomycin *strA*, *strB*, *aadA2*, *aadE*; 50-52: sulfamethoxazole *sul1-3*; 53-72: tetracycline *tetA-E*, *tetG*, *tetH*, *tetK-O*, *tetS*, *tetT*, *tetW*, *tetZ*, *tet31-34*, *tet39*; 73-75 glycopeptides *vanA*, *vanB*, *vanX*; 76-80: macrolides *ermA-F*; 81-88: streptogramins *vat*, *vatB*, *vatD-E*, *vgaA-B*, *vbgA-B*; 89-92: MRSA *spa*, *mecA*, *mecC*, *pvl*.
